# Multi-Strategy Improved Red-Billed Blue Magpie Optimization Algorithm and Its Applications

**DOI:** 10.3390/biomimetics10090592

**Published:** 2025-09-05

**Authors:** Yancang Li, Jiaqi Zhi, Xinle Wang, Binli Shi

**Affiliations:** 1 School of Civil Engineering, Hebei University of Engineering, Handan 056038, China; liyancang@hebeu.edu.cn (Y.L.); wangxl995@126.com (X.W.); shibinli2001@163.com (B.S.); 2 School of Land Science and Space Planning, Hebei GEO University, Shijiazhuang 050031, China

**Keywords:** red-billed blue magpie optimization algorithm, sinh–cosh search strategy, neighbor-guided reinforcement strategy, crossover strategy

## Abstract

To address the issues of low convergence accuracy, poor population diversity, and susceptibility to local optima in the Red-billed Blue Magpie Optimization Algorithm (RBMO), this study proposes a multi-strategy improved Red-billed Blue Magpie Optimization Algorithm (SWRBMO). First, an adaptive T-distribution-based sinh–cosh search strategy is used to enhance global exploration and speed up convergence. Second, a neighborhood-guided reinforcement strategy helps the algorithm avoid local optima. Third, a crossover strategy is also introduced to improve convergence accuracy. SWRBMO is evaluated on 15 benchmark functions selected from the CEC2005 test suite, with ablation studies on 12 of them, and further validated on the CEC2019 and CEC2021 test suites. Across all test sets, its convergence behavior and statistical significance are analyzed using the Wilcoxon rank-sum test. Comparative experiments on CEC2019 and CEC2021 demonstrate that SWRBMO achieves faster convergence and higher accuracy than RBMO and other competitive algorithms. Finally, four engineering design problems further confirm its practicality, where SWRBMO outperforms other methods by up to 99%, 38.4%, 2.4%, and nearly 100% in the respective cases, highlighting its strong potential for real-world engineering applications.

## 1. Introduction

Optimization problems involve selecting the optimal solution from a set of candidates [1], and they are widely applied to address the growing demands of modern economic and industrial development. With advancements in science and technology, real-world optimization problems have become more complex, often characterized by high dimensionality, non-differentiability, non-convexity, and computationally expensive objective functions. These characteristics make traditional optimization methods ineffective or impractical in many cases. In contrast, metaheuristic algorithms, due to their simplicity, ease of implementation, and robustness, have emerged as a promising alternative and have successfully been applied in areas such as robot navigation [2], workshop job scheduling [3], neural network adjustment [4], and feature selection [5].

Metaheuristic algorithms can be broadly categorized into four types based on their sources of inspiration: swarm intelligence behavior-based algorithms, biological evolution-based algorithms, physical law-based algorithms, and human behavior-based algorithms. Among these, swarm intelligence algorithms, as a core subclass of metaheuristic algorithms, have been widely recognized for their ability to efficiently and accurately solve complex optimization problems without relying on global information. Representative examples include Differential Evolution (DE) [6], Particle Swarm Optimization (PSO) [7], Sparrow Search Algorithm (SSA) [8], Grey Wolf Optimizer (GWO) [9], Harris Hawks Optimization (HHO) [10], Butterfly Optimization Algorithm (BOA) [11], Black Widow Optimization (BWO) [12], and Whale Optimization Algorithm (WOA) [13]. With the growing research interest in optimization, improving traditional algorithms to enhance their performance has become a major focus. For instance, Mingjun Ye et al. [14] proposed a Multi-strategy Enhanced Dung Beetle Optimization algorithm (MDBO). This algorithm replaces the original random initialization with Latin Hypercube Sampling (LHS) [15]—a typical initialization optimization strategy that enables more uniform sampling of the solution space, which helps MDBO demonstrate superior performance in terms of optimization accuracy, stability, and convergence speed. Similarly, Fu Hua et al. [16] introduced a multi-strategy enhanced Sparrow Search Algorithm, integrating an elite-driven chaotic reverse learning mechanism for population initialization and follower position refinement. These examples demonstrate the effectiveness of augmenting swarm intelligence algorithms by integrating novel improvement strategies, providing valuable insights for further improvements.

The Red-billed Blue Magpie Optimization algorithm (RBMO) [17], proposed in 2024 by Shengwei Fu and Ke Li et al., is a novel swarm intelligence optimization algorithm inspired by the cooperative and efficient hunting behaviors of red-billed blue magpies in nature. It stands out due to its strong adaptability, few parameters, and biologically grounded foundation. Unlike traditional optimization algorithms, RBMO uses a dynamic coordination mechanism that adjusts search behavior based on environmental cues, allowing it to adapt to complex and changing problem spaces. Leveraging this unique mechanism, RBMO has demonstrated promising performance in addressing complex engineering optimization tasks, such as polymer electrolyte membrane fuel cell modeling [18], antenna parameter optimization [19], and unmanned aerial vehicle (UAV) path planning [20].

Like many swarm intelligence optimization algorithms, RBMO still exhibits non-negligible limitations when addressing complex optimization problems: it suffers from relatively weak global search ability, tends to get trapped in local optima, and its final accuracy is often compromised in later iterations due to the loss of population diversity. According to the No Free Lunch theorem [21], no single algorithm performs best across all problems, which has motivated continuous efforts to enhance or hybridize existing methods. Meanwhile, it should be acknowledged that some metaheuristic algorithms outside the swarm intelligence family, such as the Survival of the Fittest Algorithm (SoFA) [22], have been proven to achieve global convergence under certain theoretical conditions. Nevertheless, within the domain of swarm intelligence optimization, practical improvements that balance exploration, exploitation, and population diversity remain a key research focus. Therefore, many scholars remain committed to proposing new algorithms or improving existing ones to enhance their applicability and optimization performance in real-world scenarios.

To address the aforementioned limitations of RBMO, this paper proposes a multi-strategy improved Red-billed Blue Magpie Optimization Algorithm (SWRBMO). By synergistically integrating three complementary strategies, SWRBMO targets and addresses the major shortcomings of RBMO in a systematic manner, which are detailed below:

Adaptive T-distribution-Based Sinh–Cosh Search Strategy: The sinh and cosh operators are introduced to inherently coordinate exploration and exploitation, effectively enhancing global search capability. It further integrates an adaptive T-distribution perturbation mechanism, which transitions from large to small perturbations over iterations, and further improves the balance between global exploration and local exploitation, directly accelerating the convergence speed.

Neighborhood-Guided Reinforcement Strategy: This strategy leverages information from neighboring individuals to guide the mutation process, generating diverse candidate solutions and strengthening the algorithm’s ability to explore unknown regions, thus improving its capability to escape from local optima.

Crossover Strategy: This mechanism introduces crossover perturbations among individuals and enables high-quality information exchange, thereby preserving population diversity in the late optimization stage and improving the final optimization accuracy.

To further clarify the innovation of the proposed SWRBMO, Table 1 systematically compares relevant algorithms from multiple perspectives, including inspiration sources, main strategies, strengths and limitations. Specifically, this paper selects classic swarm intelligence algorithms (DE, PSO, and SSA), the original RBMO, and the recently developed representative multi-strategy improved algorithm MDBO as comparison objects.

The structure of this paper is as follows: Section 2 describes the optimization process of the RBMO algorithm. Section 3 introduces the SWRBMO algorithm in detail, including the integration of the proposed strategies and a complexity analysis. Section 4 presents extensive experimental results on benchmark functions and the CEC2019 and CEC2021 test suites, along with ablation studies and comparative analysis. Section 5 demonstrates its successful application to four real-world engineering optimization problems: the robot gripper optimization problem, the industrial refrigeration system optimization problem, the reinforced concrete beam design optimization problem, and the step cone pulley problem. The optimization results show that SWRBMO’s overall performance is 99%, 38.4%, 2.4% and nearly 100% higher than that of the comparative algorithms for the four problems, respectively. This confirms SWRBMO’s strong potential for engineering applications. Section 6 concludes the study and discusses future directions.

## 2. Red-Billed Blue Magpie Optimization Algorithm

The RBMO algorithm [17] is a novel swarm intelligence optimization algorithm inspired by the adaptive foraging strategies of red-billed blue magpies, which dynamically adjust their population size and search patterns based on environmental resources. To enhance its performance, in this study, a population balance coefficient α (set to 0.5) is introduced to dynamically coordinate the algorithm’s exploration and exploitation.

### 2.1. Initial Population

In the RBMO algorithm, a population consisting of *n* individuals is randomly initialized in a D-dimensional search space. Each individual’s position is uniformly sampled within the predefined upper and lower bounds, which are defined as follows:

(1) $$X_{ij}=(ub-lb)\times {rand}_{1}+lb$$

where $X_{ij}$ represents the coordinate of the *i*-th red-billed blue magpie in the *j*-th variable. ub and lb represent the upper and lower bounds of the search space, respectively, and ${rand}_{1}\;$ is a uniformly distributed random number within the interval [0, 1].

### 2.2. Search for Food

In natural settings, red-billed blue magpies employ various strategies to obtain food resources, such as hopping, walking on the ground, or probing tree branches. These birds tend to forage in either small groups (usually 2 to 5) or large groups (10 or more). This behavioral flexibility enables the magpies to dynamically adjust their foraging tactics in response to changes in environmental conditions and food availability. Inspired by this adaptive foraging strategy, the RBMO algorithm simulates both small-group and large-group search behaviors to enhance its exploration capabilities in the solution space.

The math for foraging in small groups is as follows:

(2) $$X_{i}^{T+1}=X_{i}^{T}+\left(\frac{1}{G_{s}}\times \sum_{m=1}^{G_{s}}X_{m}^{T}-X_{r}^{T}\right)\times {rand}_{2}$$

The math for foraging in large groups is as follows:

(3) $$X_{i}^{T+1}=X_{i}^{T}+\left(\frac{1}{G_{l}}\times \sum_{m=1}^{G_{l}}X_{m}^{T}-X_{r}^{T}\right)\times {rand}_{3}$$

where $X_{i}^{T+1}$ and $X_{i}^{T}$ denote the updated and current positions, respectively. The variables $G_{s}\in \left[2,\;5\right]$ and $G_{l}\in \left[10,\;n\right]$ represent the group sizes for small and large groups, respectively. The vector $X_{m}^{T}$ denotes the position of the *m*-th magpie selected at random from the population, whereas $X_{r}^{T}\;$ refers to the location of another randomly chosen individual during the current iteration. The variables ${rand}_{2}$ and ${rand}_{3}$ are uniformly distributed within [0, 1].

The combined model is expressed as

(4) $$X_{i}^{T+1}=\left\{\begin{array}{c} X_{i}^{T}+\left(\frac{1}{G_{s}}\times \sum_{m=1}^{G_{s}}X_{m}^{T}-X_{r}^{T}\right)\times {rand}_{2},\;\text{if}\;rand<\alpha \\ X_{i}^{T}+\left(\frac{1}{G_{l}}\times \sum_{m=1}^{G_{l}}X_{m}^{T}-X_{r}^{T}\right)\times {rand}_{3},\;\text{else} \end{array}\right.$$

### 2.3. Attacking Prey

When attacking prey, red-billed blue magpies exhibit efficient abilities and collaborative behaviors. Depending on the characteristics of the prey, they will adopt diversified hunting strategies, such as rapid pecking, flexible jumping and aerial interception. When operating in small groups (usually 2 to 5), magpies primarily target small plants and animals. In contrast, when forming large groups (10 or more), individuals collaborate closely to pursue larger prey, including sizable insects or small vertebrates. This flexible use of multiple cooperative strategies significantly enhances hunting efficiency. The corresponding mathematical model for small-group hunting behavior is formulated as follows:

(5) $$X_{i}^{T+1}=X_{food}^{T}+CF\times \left(\frac{1}{G_{s}}\times \sum_{m=1}^{G_{s}}X_{m}^{T}-X_{i}^{T}\right)\times {randn}_{1}$$

where $X_{food}^{T}$ indicates the location of the food source, which indicates the current optimal solution. The variable ${randn}_{1}$ represents a random value drawn from a standard normal distribution, characterized by a mean of 0 and a standard deviation of 1.

CF is the step control factor (from the original literature [17]), which is mathematically represented as follows:

(6) $$CF={\left(1-\left(\frac{t}{T}\right)\right)}^{\left(2\times \frac{t}{T}\right)}$$

where t represents the current iteration number, and T represents the maximum iteration number.

The mathematical representation when acting in large-group hunting behavior is as follows:

(7) $$X_{i}^{T+1}=X_{food}^{T}+CF\times \left(\frac{1}{G_{l}}\times \sum_{m=1}^{G_{l}}X_{m}^{T}-X_{i}^{T}\right)\times {randn}_{2}$$

where ${randn}_{2}$ represents a standard normally distributed random number with a mean of 0 and a standard deviation of 1.

The whole stage of attacking prey is mathematically represented as follows:

(8) $$X_{i}^{T+1}=\left\{\begin{array}{c} X_{food}^{T}+CF\times \left(\frac{1}{G_{s}}\times \sum_{m=1}^{G_{s}}X_{m}^{T}-X_{i}^{T}\right)\times {randn}_{1},\;\text{if}\;rand<\alpha \\ X_{food}^{T}+CF\times \left(\frac{1}{G_{l}}\times \sum_{m=1}^{G_{l}}X_{m}^{T}-X_{i}^{T}\right)\times {randn}_{2},\;\text{else} \end{array}\right.$$

### 2.4. Food Storage

In addition to foraging and hunting, the red-billed blue magpies also exhibit a unique food storage habit. They conceal surplus food in hidden locations, effectively creating an emergency food reserve. This storage strategy ensures a stable energy supply during times of food scarcity. In the context of algorithmic modeling, this characteristic of efficiently preserving data aids the algorithm in progressively approaching the global optimum during the solution process. The mathematical expression of this behavior is as follows:

(9) $$X_{i}^{T+1}=\left\{\begin{array}{c} X_{i}^{T},\;\text{if}\;{fitness}_{i}^{old}>{fitness}_{i}^{new}\\ X_{i}^{T+1},\;\text{else}\; \end{array}\right.$$

where ${fitness}_{i}^{old}$ denotes the fitness value before the position update, while ${fitness}_{i}^{new}$ represents the fitness values after the update.

## 3. Improved Optimization Algorithm for Red-Billed Blue Magpie

### 3.1. Adaptive T-Distribution-Based Sinh–Cosh Search Strategy

The original RBMO adopts a monotonic perturbation mechanism for position updating. While this mechanism ensures stability, it has two major drawbacks: first, the weak information exchange among individuals reduces global exploration ability; second, its fixed disturbance pattern lacks adaptability in later iterations, which slows down convergence and makes it difficult to handle dynamic and complex search landscapes. These limitations motivate the introduction of a more flexible strategy to address the aforementioned shortcomings.

In recent years, a number of mathematical optimization techniques—such as the Sine–Cosine Algorithm (SCA) [23] and the Arithmetic Optimization Algorithm (AOA) [24]—have opened new directions for the development of metaheuristic strategies. Within this context, the Sinh–Cosh Optimizer (SCHO) [25] is particularly noteworthy, as its distinctive mathematical characteristics provide promising potential to overcome the limitations of RBMO, especially in strengthening global exploration and improving adaptability across diverse search landscapes. To further introduce randomness, enhance search dynamics, and relax the rigidity of RBMO’s original monotonic perturbation mechanism, a uniformly distributed random variable $k_{1}$ (within the range [0, 1]) is integrated into the improved strategy. The mathematical basis of the enhancement lies in the sinh and cosh functions, which are defined as follows:

(10) $$sinh={(e}^{k1}-e^{-k1})/2$$

(11) $$cosh={(e}^{k1}+e^{-k1})/2$$

To regulate the adjustment intensity, an intermediate control variable c is designed as

(12) c=3×(−1.3×(−t/T)+0.45)

The update factor w, which determines the scale of position adjustment, is defined as

(13) $$w=w_{1}\times c\times (cosh+\mu \times sinh-1)$$

The critical parameters in these formulas are inherited from the validated settings of SCHO, ensuring both theoretical consistency and empirical reliability. Specifically, the constant term 0.45 in Formula (12) and the sensitivity coefficient μ (commonly set to 0.388) in Formula (13) follow standard configurations reported in [25], rather than being tuned arbitrarily. In addition, $w_{1}$ is a uniformly distributed random number in [0, 1]. Notably, the cosh function is always greater than 1, enhancing the exploration capability, while the sinh function lies within [−1, 1], contributing to controlled perturbations. These complementary effects improve the balance between exploration and exploitation. Based on these insights, the sinh–cosh search strategy is incorporated into RBMO to overcome its inherent limitations.

Based on the balance coefficient α, two distinct update rules are applied:

(14) $$X_{new}^{T}=\left\{\begin{array}{c} X_{i}^{T}+\left(\frac{1}{G_{s}}\times \sum_{m=1}^{G_{s}}X_{m}^{T}-w\times X_{r}^{T}\right)\times {rand}_{2},\;if\;rand<\alpha \\ X_{i}^{T}+\left(\frac{1}{G_{l}}\times \sum_{m=1}^{G_{l}}X_{m}^{T}-w\times X_{r}^{T}\right)\times {rand}_{3},\;else \end{array}\right.$$

where $X_{new}^{T}$ denotes the updated position, while $X_{i}^{T}$ refers to its current location in the search space. The parameters $G_{s}\in \left[2,\;5\right]$ and $G_{l}\in \left[10,\;n\right]$ define the sizes of small and large groups. Although this sinh–cosh search strategy enriches the exploration–exploitation balance, its convergence speed can still be limited. To further address this issue, an adaptive T-distribution strategy is proposed. Its mathematical representation is as follows:

(15) $$X_{new}^{T}=\left\{\begin{array}{c} trnd\left(freen\right)\times X_{i}^{T}+\left(\frac{1}{G_{s}}\times \sum_{m=1}^{G_{s}}X_{m}^{T}-X_{r}^{T}\right)\times {rand}_{2},\;if\;rand<\alpha \\ trnd\left(freen\right)\times X_{i}^{T}+\left(\frac{1}{G_{l}}\times \sum_{m=1}^{G_{l}}X_{m}^{T}-X_{r}^{T}\right)\times {rand}_{3},\;else \end{array}\right.$$

where trnd(freen) is a random number generated according to the adaptive T-distribution, which introduces the fluctuation characteristics of this distribution into the algorithm. For the adaptive perturbation, a dynamic parameter freen is introduced. Here, freen denotes the degree of freedom of the T-distribution (the “free-n” parameter), and its mathematical expression is defined as follows:

(16) $$freen=e^{3{\left(t/T\right)}^{2}}$$

**Figure 1:**
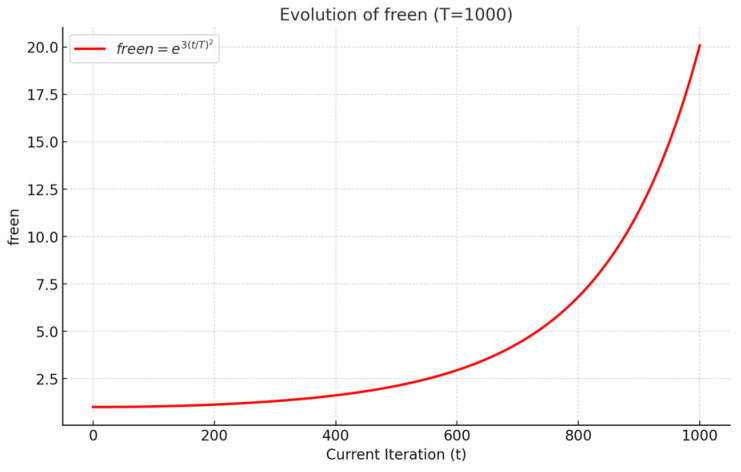
Iteration variation curve of T-distribution perturbation.

During algorithm iteration, the T-distribution’s degrees of freedom adaptively change with the ratio of current iteration t to total iterations T. Figure 1 shows the T-distribution perturbation curve across iterations. As illustrated, this formulation yields a heavy-tailed distribution during early iterations (when t/T is small), facilitating escape from local optima and global exploration (compensating for RBMO’s original monotonic disturbance’s insufficient exploration). As iterations progress and t/T → 1, the T-distribution gradually approximates a normal distribution, enabling more refined local search around promising regions and effectively accelerating convergence (addressing the slow convergence issue of the original algorithm).

Finally, the adaptive T-distribution strategy is integrated with the sinh–cosh search strategy to construct a more robust position update rule:

(17) $$X_{new}^{T}=trnd\left(freen\right)\times X_{i}^{T}+\left(\frac{1}{G_{s}}\times \sum_{m=1}^{G_{s}}X_{m}^{T}-w{\;\times \;X}_{r}^{T}\right)\times {rand}_{2},\;if\;rand<\alpha$$

(18) $$X_{new}^{T}=trnd\left(freen\right)\times X_{i}^{T}+\left(\frac{1}{G_{l}}\times \sum_{m=1}^{G_{l}}X_{m}^{T}-w\times X_{r}^{T}\right)\times {rand}_{3},\;else$$

In summary, the sinh–cosh search strategy introduces diversity and achieves a balance between exploration and exploitation, while the adaptive T-distribution introduces dynamic perturbations shifting from global to local focus. Their combination complements RBMO’s original monotonic perturbation, enhancing global optimization and convergence speed.

### 3.2. Neighborhood-Guided Reinforcement Strategy

In the standard RBMO, individuals are strongly guided by the current global best solution. However, this reliance makes the algorithm prone to premature convergence: as iterations progress, individuals crowd around the same region, diversity diminishes, and the search stagnates. To address this limitation, a neighborhood-guided reinforcement strategy inspired by [26] is introduced. Instead of focusing solely on the global best, this mechanism incorporates information from neighboring individuals, thereby promoting local interactions and generating a richer set of candidate solutions. This localized interaction introduces richer candidate solutions, maintains population diversity, and enhances the ability to escape local optima.

Governed by the balance coefficient α, the position update rule of this neighborhood-guided reinforcement strategy is formulated as follows:

(19) $$X_{new}^{T}=X_{i}^{T}+rand\left(1,1\right)\times \left(X_{i}^{Best}-I\times X_{i}^{near}\right),\;if\;rand<\alpha$$

(20) $$X_{new}^{T}=X_{food}^{T}+CF\times \left(\frac{1}{G_{l}}\times \sum_{m=1}^{G_{l}}X_{m}^{T}-X_{i}^{T}\right)\times {randn}_{2\;},\;else$$

where $X_{i}^{Best}$ is the optimal position of the i-th individual and $X_{i}^{near}$ is the neighboring individual of the current position. When i≠1, the i−1 individual is considered as the neighboring individual; when i=1, the first individual itself is taken as the neighboring individual. I is a randomly generated integer within {1, 2}. Additionally, ${randn}_{2}$ is a random number following the standard normal distribution with a mean of 0 and a standard deviation of 1.

### 3.3. Crossover Strategy

As iterations increase, red-billed blue magpies tend to congregate around the current optimal solution. This clustering behavior reduces population diversity, which restricts the search capability and reduces convergence accuracy. To address the loss of diversity and declining convergence performance during iterations, this paper introduces an innovative crossover strategy [27] into the RBMO algorithm. This strategy improves search efficiency by applying crossover operations that maintain diversity and enhance convergence accuracy.

The crossover strategy consists of two components: horizontal crossover and vertical crossover. Horizontal crossover allows individuals in the population to exchange information comprehensively, promoting extensive exploration of the solution space and enhancing global search capability. Vertical crossover performs crossover operations between different dimensions within an individual, effectively preventing the algorithm from becoming trapped in local optima in certain dimensions. Through the inherent randomness and diversity of these crossover operations, the algorithm continuously produces novel solutions. When the population is trapped in local optima, these new solutions provide alternative search directions, increasing the probability of escaping stagnation and progressing toward the global optimum. After each crossover, offspring compete with their parents, and the individuals with superior fitness are retained, ensuring both diversity maintenance and convergence accuracy.

#### 3.3.1. Horizontal Crossing

In each iteration, pairs of individuals are randomly matched to perform arithmetic crossover across all dimensions. Given parent individuals $X_{i}$ and $X_{j}$, the offspring are generated as follows:

(21) $$M_{i,d}^{hc}=\alpha_{1}\times X_{i}\left(d\right)+\left(1-\alpha_{1}\right)\times X_{j}\left(d\right)+q_{1}\times \left(X_{i}\left(d\right)-X_{j}\left(d\right)\right)$$

(22) $$M_{j,d}^{hc}=\alpha_{2}\times X_{j}\left(d\right)+\left(1-\alpha_{2}\right)\times X_{i}\left(d\right)+q_{1}\times \left(X_{i}\left(d\right)-X_{j}\left(d\right)\right)$$

where $\alpha_{1}$ and $\alpha_{2}$ are random cross coefficients between [0, 1], and $q_{1}$ and $q_{2}$ are random numbers between [0, 1]. $X_{i}\left(d\right)$ and $X_{j}\left(d\right)$ are parents of $X_{i}$ and $X_{j}$, respectively. $M_{i,d}^{hc}$ and $M_{j,d}^{hc}$ are D-dimensional descendants of $X_{i}\left(d\right)$ and $X_{j}\left(d\right)$, respectively. After crossover, the fitness of offspring is compared to that of their parents, and the individuals with higher fitness are preserved.

#### 3.3.2. Vertical Crossing

Vertical crossover is an arithmetic crossover applied between different dimensions within a single individual. This operation recombines information across two randomly selected dimensions $d_{1}$ and $d_{2}$, preventing the algorithm from getting stuck in local optima restricted to specific dimensions. After normalizing $d_{1}$ and $d_{2}$, the offspring are updated as follows.

(23) $$M_{i,d1}^{vc}=\beta \times X_{i}\left(d_{1}\right)+\left(1-\beta \right)\times X_{j}\left(d_{2}\right)$$

where the cross parameter β is a random number in [0, 1], and $M_{i,d1}^{vc}$ is the child of $X_{i}\;$ and $X_{j}$ in the $d_{1}$ and $d_{2}$ dimensions. After vertical crossover, offspring fitness is compared with that of their parents, and the better individuals are retained.

### 3.4. The Pseudocode of SWRBMO Algorithm

The specific implementation steps and pseudocode of the SWRBMO algorithm are shown below:

Step 1. Initialization: Set the maximum number of iterations *T*, population size *N*, problem dimension *D*, balance coefficient α, and the search boundaries lb and ub. Initialize other relevant parameters as needed. The population is then generated by randomly creating *N* individuals (magpie positions), which form the initial solution space.

Step 2. Best Solution Update: At the beginning of each iteration, evaluate the fitness of all individuals according to the objective function.

Step 3. Adaptive T-Distribution-based sinh–cosh Search Strategy: For each individual in the population, if rand < α, the position is updated using Formula (17). Otherwise, the position is updated using Formula (18).

Step 4. Neighborhood-guided Reinforcement Strategy: If rand < α, the individual’s position is updated according to Formula (19), where interactions with neighboring individuals increase diversity and reduce stagnation. Otherwise, update its position using Formula (20).

Step 5. Crossover Strategy: Perform crossover operations on the updated individuals using Formulas (21)–(23). Specifically, horizontal crossover is implemented by exchanging information between different individuals, while vertical crossover is achieved by recombining information within a single individual.

Step 6. Food Source Update: After the crossover operation, refresh the food source using Formula (9), and retain the best-performing solution in the current iteration to guide the subsequent search process.

Step 7. Iteration Update: Repeat Steps 2 to 6 continuously, incrementing the iteration count each time until the maximum number of iterations T is reached.

The pseudocode of the SWRBMO algorithm is shown in Algorithm 1.
**Algorithm 1** The pseudocode of the SWRBMO algorithm Input: The dimension *D*, maximum number of iterations *T*, and population size *N*Output: Global optimal solution Global optimal solution1:      Procedure SWRBMO2:      Initialize the key parameters *T*, *D*, *N*, *t*, and *α*3:      While *t* < *T* +14:      Calculate the position of each individual5:      Update the optimal solution6:      Exploration:7:      for *i* = 1: *N*8:         if rand < α9:         Modify the individual’s position using Equation (17)10:        else11:        Modify the individual’s position using Equation (18)12:        end if13:     Exploitation:14:        if rand < α15:        Modify the magpie’s coordinates using Equation (19)16:        else17:        Update the magpie’s location using Equation (20)18:        end if19:        Execute the Crossover Strategy using Equations (21)–(23)20:     end for21:     Refresh the food storage, using Equation (9)22:     *t* = *t* + 123:     end while24:     Return best solution

### 3.5. Time Complexity Analysis

According to the complexity analysis framework of the original RBMO algorithm, RBMO’s computational complexity depends on two core stages: the solution initialization stage (*O*(*N*), where *N* is the number of search agents) and the solution update stage (*O*(*T* × *N*) + *O*(2 × *T* × *N* × *D*), where T is the maximum number of iterations and dim is the problem dimension). The computational complexity framework of the proposed SWRBMO is consistent with that of RBMO, as it also focuses on the initialization stage and the solution update stage (with three new strategies embedded in the latter, without altering the core complexity dependence).

In the initialization stage, SWRBMO adopts the same logic as RBMO to generate *n* search agents, so its time complexity remains O(*N*), consistent with RBMO. In the solution update stage, SWRBMO retains RBMO’s original operations contributing *O*(*T* × *N*) + *O*(2 × *T* × *N* × *D*) and embeds three new strategies (adaptive T-distribution-based sinh–cosh search, neighborhood-guided reinforcement, and crossover). All new strategies operate on *n* agents across dim dimensions per iteration, contributing complexities of *O*(*T* × *N* × *D*), *O*(*T* × *N* × *D*), and *O*(1.5 × *T* × *N* × *D*), respectively. Thus, the total complexity of SWRBMO’s solution update stage is *O*(*T* × *N*) + *O*(2 × *T* × *N* × *D*) + O(*T* × *N* × *D*) + *O*(*T* × *N* × *D*) + *O*(1.5 × *T* × *N* × *D*). Merging like terms gives *O*(*T* × *N*) + *O*(5.5 × *T* × *N* × *D*). Combining the initialization stage *O*(*N*) and the solution update stage O(*T* × *N*) + *O*(5.5 × *T* × *N* × *D*), the total complexity of SWRBMO is *O*(*N*) + *O*(*T* × *N*) + *O*(5.5 × *T* × *N* × *D*).

Per the rules of complexity analysis (ignoring constant factors (5.5) and lower-order terms *O*(*N*) and *O*(*T* × *N*), which are negligible compared to the high-order term *O*(*T* × *N* × *D*), the total complexity is simplified to *O*(*T* × *N* × *D*).

Although SWRBMO integrates three strategies based on RBMO, its total computational complexity maintains the same order *O*(*T* × *N* × *D*) as the original RBMO. This confirms that the improved algorithm does not introduce significant additional computational burden while enhancing performance, ensuring its efficiency and practical applicability for solving complex optimization problems.

## 4. Algorithm Performance Test and Analysis

To comprehensively assess the effectiveness of the proposed adaptive T-distribution-based sinh–cosh search strategy, neighborhood-guided reinforcement strategy, and crossover strategy on the RBMO algorithm, as well as to systematically evaluate the SWRBMO, the experimental study was designed following a sequential manner from strategy-level validation to comprehensive performance comparison. Accordingly, the experiments were divided into two stages.

### 4.1. Experimental Design and Parameter Settings

#### 4.1.1. Division of the Experimental Stages

Validation of the individual strategies: To disentangle and assess the contribution of each improvement, comparisons were performed between the original RBMO, its single-strategy enhanced variants, and the proposed SWRBMO across 15 benchmark functions from the CEC2005 test suite. In addition, ablation experiments were conducted on 12 of these functions to further substantiate the rationale for subsequent multi-strategy integration.

Comprehensive performance comparison: After validating the effectiveness of the individual strategies, the proposed SWRBMO was benchmarked against both classical and recently developed metaheuristic algorithms using the CEC2019 and CEC2021 test suites. Compared to CEC2005, these two suites incorporate more intricate function constructions and thus present more challenging optimization landscapes, making them well suited for a rigorous assessment of algorithmic performance. To further enhance statistical reliability, the Wilcoxon rank-sum test was applied across all three test suites (CEC2005, CEC2019, and CEC2021). The inclusion of CEC2005 not only provides a widely recognized historical reference but also complements the analysis, ensuring that the conclusions regarding SWRBMO’s performance are comprehensive and statistically robust.

This study selected the CEC2005, CEC2019, and CEC2021 test suites. The core objective was to move from single-strategy validation to comprehensive performance comparison. The two-stage experimental design formed a complementary and mutually supportive relationship.

#### 4.1.2. Experimental Parameters and Environment

To ensure fairness and eliminate parameter-related bias, all experiments adopted the same settings: population size *N* = 30, and the maximum number of iterations *T* = 1000. Each algorithm was independently run 30 times to mitigate stochastic effects. Experiments were carried out on a 64-bit Windows 10 system equipped with an Intel Core i7-8750H CPU, 16 GB RAM, and MATLAB 2022b.

### 4.2. Effectiveness Analysis of the Improvement Strategy

#### 4.2.1. Selection of Test Functions

To verify the fundamental accuracy of the proposed SWRBMO, the CEC2005 test suite (see Table 2) was employed. CEC2005 is one of the most canonical and widely cited benchmark sets in swarm intelligence research, and its availability of known optima makes it particularly suitable for error-to-optimum accuracy verification. Although other benchmarks such as GKLS (ND type) [28] can generate test classes with controllable global minima, it has been less frequently adopted in recent studies. In contrast, CEC2005 enables fair and direct comparison with a large body of related work, which is crucial for consistency and benchmarking validity.

The selected 15 functions cover three representative categories: F1–F7 are unimodal functions, which are used to assess the algorithm’s exploitation capability and convergence speed. Functions F8–F13 are multimodal, containing numerous local optima, and are commonly used to evaluate the algorithm’s global search ability and robustness against local entrapment. F14 and F15 are composite multimodal problems with fixed dimensions, representing more complex and challenging optimization scenarios.

For CEC2005, F1–F13 were tested under fixed dimensions of 30 or 100, while F14–F15 followed their inherent dimensionality. This controlled setting eliminates dimensional fluctuations, ensuring that the evaluation focuses on the strategies themselves and rigorously validates stability and robustness across diverse function types.

#### 4.2.2. Effectiveness Analysis of Different Improvement Strategies

**Figure 2:**
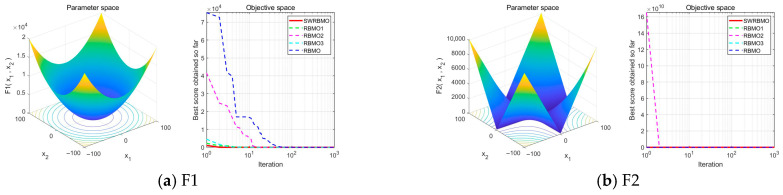

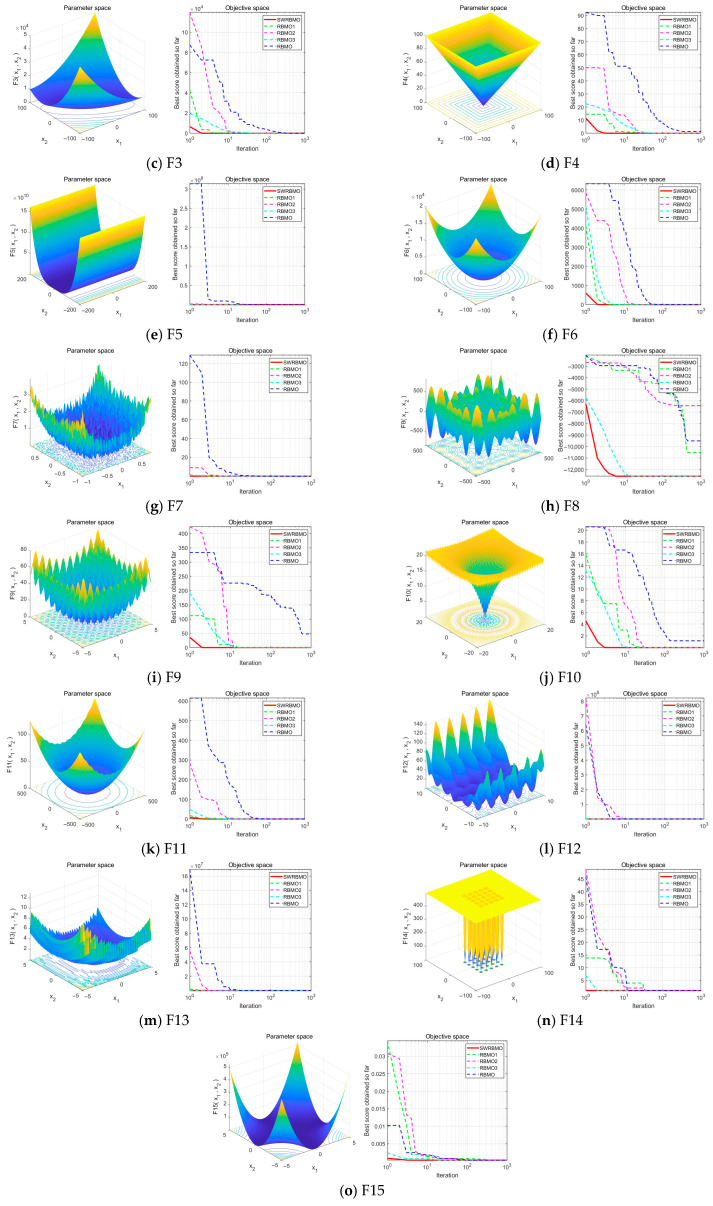
Comparison of convergence curves of different improvement strategies.

To rigorously assess the effectiveness of the proposed SWRBMO algorithm, three enhanced variants of RBMO were developed: RBMO1, incorporating the adaptive T-distribution-based sinh–cosh search strategy; RBMO2, integrating the neighborhood-guided reinforcement strategy; and RBMO3, embedding the crossover strategy. Comparative experiments were conducted among the original RBMO, its three single-strategy variants, and the full SWRBMO across 15 benchmark functions with diverse characteristics. The numerical optimization results are reported in Table 3 and Table 4, while the convergence behaviors under the 30-dimensional setting are depicted in Figure 2.

Under the 30-dimensional setting, the evaluation on unimodal functions (F1–F7) reveals that all strategy-enhanced variants (RBMO1, RBMO2, and RBMO3) markedly outperform the original RBMO in terms of optimal values, mean values and standard deviations. Among them, SWRBMO consistently exhibits the most competitive overall performance, achieving faster convergence, superior optima, improved mean results, and reduced standard deviations. Specifically, for functions F1–F4, SWRBMO, RBMO1, and RBMO2 all attain the theoretical optima across all metrics, while RBMO3 also demonstrates significant improvements over the baseline. On the more challenging F5, SWRBMO’s best value and standard deviation are slightly lower than RBMO3 and RBMO2, respectively, yet it still outperforms RBMO—reflecting the effectiveness of the integrated strategies. For F6, both SWRBMO and RBMO3 reach the theoretical optimum, highlighting the strong utility of the crossbar strategy in complex cases. Although SWRBMO does not achieve the global optimum on F7, it nevertheless surpasses RBMO across all indicators.

The results on multimodal functions (F8–F13) further corroborate the efficacy of the proposed strategies. For F8, F12, and F13, SWRBMO and RBMO3 exhibit comparable performance, indicating that the crossbar strategy is instrumental in escaping local optima. For F9–F11, SWRBMO, RBMO1, and RBMO2 reach the theoretical optima, while RBMO3 achieves the optima on F9 and F11 and closely approaches it on F10. Collectively, these findings substantiate that the integrated multi-strategy framework of SWRBMO substantially enhances both convergence efficiency and global exploration capability.

For the fixed-dimensional multimodal functions F14 and F15, the algorithm achieves a small deviation between the best and mean results, indicating its potential applicability in more complex and challenging optimization scenarios.

When the dimension is expanded to 100, although there are slight differences in the optimal values, mean values and standard deviations among various functions, SWRBMO can generally achieve better optimal values, average values and stability indicators in most cases. This indicates that the proposed strategy remains effective and stable even under high-dimensional conditions.

Further analysis of the algorithm’s stability is conducted by examining the convergence curves presented in Figure 2. The iterative patterns across the benchmark functions exhibit a high degree of similarity. In particular, the SWRBMO algorithm, enhanced with multiple strategies, demonstrates nearly linear convergence trajectories on functions F1–F3, F5, F7, and F11–F15, rapidly approaching the optimal solution. This behavior highlights the algorithm’s strong global optimization capability and fast convergence speed.

Similarly, the RBMO3 algorithm, incorporating the crossbar strategy, also exhibits nearly straight-line convergence on functions F1, F2, F5, F7, and F11–F15. This result further validates that the crossover mechanism effectively accelerates convergence and improves solution quality. For functions F4, F6, F8, F9, and F10, the convergence curves follow a steep, approximately linear descent, with fitness values dropping sharply as iterations progress. This pattern indicates the algorithm’s ability to quickly approach near-optimal solutions while effectively avoiding local optima.

### 4.3. Ablation Study

To rigorously evaluate the individual contributions and potential synergistic effects of the three optimization strategies incorporated into the SWRBMO algorithm, an ablation study was carried out using the first 12 benchmark functions from the CEC2005 test suite. Four algorithmic settings were considered: the baseline RBMO and three ablation variants, each obtained by removing a single strategy—BRBMO (excluding the crossover strategy), MRBMO (excluding the neighborhood-guided reinforcement strategy), and NRBMO (excluding the adaptive T-distribution-based sinh–cosh search strategy). All experiments in the ablation study were conducted with the problem dimension set to 30.

As shown in Table 5, SWRBMO consistently outperforms or matches the performance of other algorithms across most test functions, demonstrating strong solution accuracy and robustness. In contrast, RBMO exhibits the weakest overall performance.

Performance across function types: For the unimodal functions (F1–F7), all algorithms except RBMO reach theoretical optima on F1–F4, indicating limited strategy impact in low-complexity scenarios. However, BRBMO shows significant increases in average error and standard deviation on F5–F7, highlighting vulnerabilities of single-strategy designs in moderately complex cases. For the multimodal functions (F8–F12), performance gaps widen. BRBMO suffers severe degradation with high variability on F8 and F12, underscoring the critical role of crossover strategies in maintaining convergence stability. SWRBMO, along with NRBMO and BRBMO, achieves theoretical or near-optimal results on multiple functions with minimal standard deviations, confirming the synergistic effects of the strategy combinations.

Insights from ablation studies (Table 5): RBMO1 (with only the T-distribution strategy) maintains stable performance across most functions. However, it shows mediocre results on F6 and F12 due to insufficient exploration, leading to local optima and breaks through this bottleneck when combined with the neighborhood strategy. RBMO2 (with only the neighborhood-guided reinforcement strategy) shows limited performance on F2, F4, F5–F8, and F12, but exhibits significant improvements in all metrics when combined with other strategies. RBMO3 (with only the crossover strategy) performs poorly on F2–F5, F7, F10, and F11 but sees a sharp increase in performance after integrating additional strategies. This highlights the core value of ablation experiments: dissecting single-strategy bottlenecks through controlled variables, quantifying the contribution of each strategy, and providing robust evidence for the necessity of multi-strategy integration.

In summary, the integration of multiple strategies significantly enhances the overall performance of the algorithm, underscoring the inherent advantages of complementarity and synergy among the strategies. The ablation study, conducted via controlled variable analysis and comparative evaluation, validates the effectiveness of the proposed strategy combination in solving complex optimization problems.

**Figure 3:**
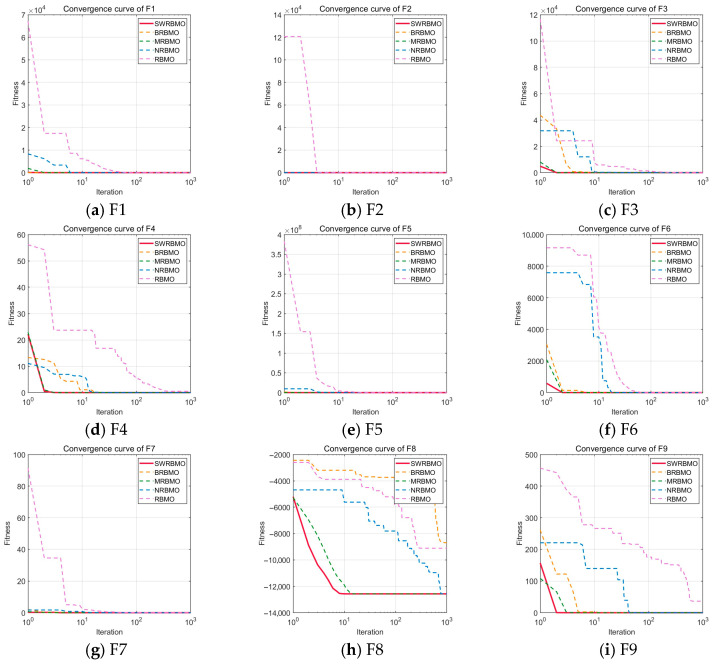

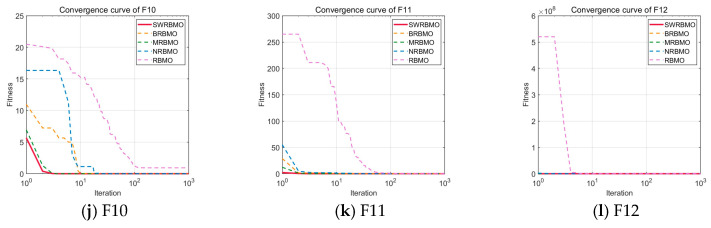
Iteration curves of the ablation study.

Based on the convergence curves of F1–F12 in Figure 3, the SWRBMO algorithm shows a clear advantage. It converges to near-optimal values faster than other methods in most cases and maintains low fluctuations after convergence, indicating excellent stability. In contrast, the ablation algorithms generally show slower convergence, lower accuracy, and less stability than SWRBMO.

The ablation results confirm the soundness of the algorithm design in two ways. First, removing any strategy leads to performance degradation. Only the combined use of multiple strategies improves convergence speed, accuracy, and stability. Second, the ablation tests reveal the weaknesses of individual strategies. NRBMO (without the adaptive T-distribution-based sinh–cosh search strategy) has poor early exploration on unimodal functions. BRBMO (without the crossover strategy) tends to get stuck in local optima on multimodal functions. These results highlight the unique roles of each strategy. The adaptive T-distribution-based sinh–cosh search strategy enhances global exploration, while the crossover strategy maintains population diversity and prevents premature convergence. Together, they create a cooperative mechanism that compensates for the shortcomings of single strategies. This further confirms the importance and necessity of multi-strategy collaboration in the SWRBMO algorithm.

### 4.4. Comparison and Analysis of SWRBMO Optimization Results with Other Optimization Algorithms

To further evaluate the optimization ability and robustness of the proposed SWRBMO algorithm, the CEC2019 and CEC2021 benchmark test suites were adopted. For comparative analysis, three classical metaheuristics, two recently developed optimization algorithms, and three advanced improved algorithms are included, namely the Whale Optimization Algorithm (WOA) [13], Pelican Optimization Algorithm (POA) [29], Harris Hawks Optimization (HHO) [10], Superb Fairy-wren Optimization Algorithm (SFOA) [30], Snow Geese Algorithm (SGA) [31], Golden Sine-based Average Best Optimizer (GSABO) [32], improved Golden Jackal Optimization Algorithm (IWKGJO) [33] and Multi-Strategy Chimp Optimization Algorithm (EOSMICOA) [34]. The problem dimensionality is set to 10 for CEC2019 and 20 for CEC2021, thereby enabling evaluation under both low- and medium-dimensional scenarios.

Results presented in Table 6 and Table 7 demonstrate that SWRBMO consistently achieves superior performance across the majority of functions. Furthermore, a comparative analysis with several other algorithms shows that SWRBMO achieves superior performance, further validating its effectiveness and practicality.

#### 4.4.1. Performance Analysis Using the CEC2019 Test Function Suite

As shown in Table 6, the SWRBMO algorithm overall demonstrates better optimization performance than the other eight advanced algorithms. Notably, the theoretical global optimal value of the functions in the CEC2019 test function suite is 1. Specifically, SWRBMO uniquely achieves the theoretical global optimal value of 1 on GF1 and GF3, indicating excellent search capability. For functions GF2, GF8, and GF9, its best and average fitness values are closer to the theoretical optimal values, highlighting stronger global search ability and higher precision in solving medium-difficulty problems. For GF4, although SWRBMO’s best value, average value, and standard deviation are slightly higher than those of IWKGJO, its overall performance still surpasses most other algorithms, reflecting good stability. On GF5 and GF7, SWRBMO achieves more stable results with lower average and standard deviation values. On GF6, the algorithm’s optimal value and standard deviation are inferior to those of IWKGJO and GSABO, respectively, but it shows a better average value. For function F10, IWKGJO achieves the best optimal value and average value, while SWRBMO demonstrates a more favorable standard deviation.

Although some algorithms perform better on individual functions, SWRBMO shows more consistent advantages across most test functions. In summary, SWRBMO exhibits significant advantages in global search ability, convergence speed, and optimization precision. These results further confirm the effectiveness of its multi-strategy collaborative design.

**Figure 4:**
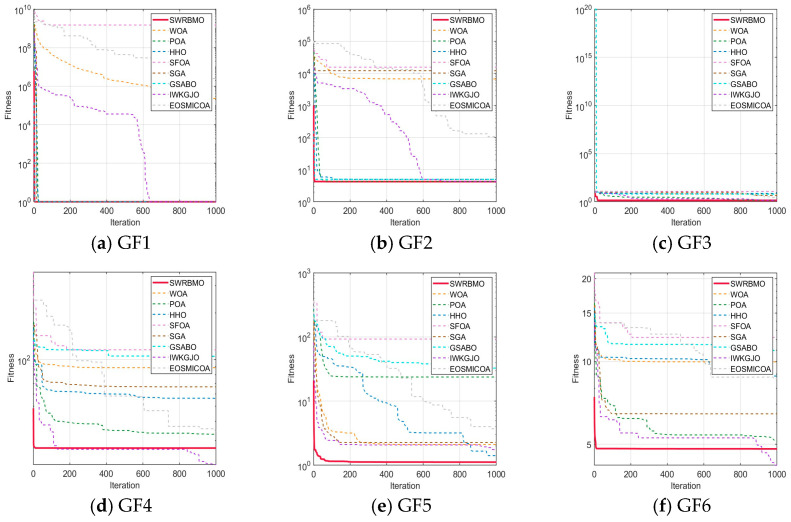

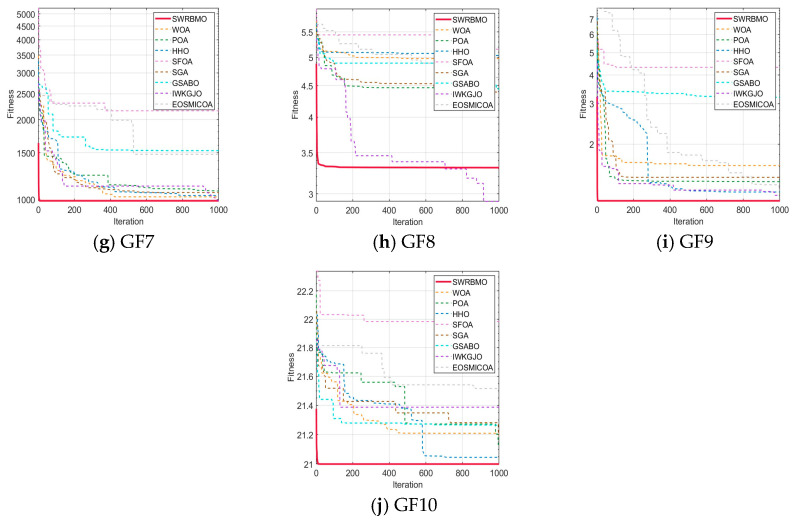
Curve comparison with other optimization algorithms on CEC2019.

As shown in Figure 4, the SWRBMO algorithm demonstrates clear advantages in convergence speed, solution accuracy, and the quality of the obtained optimal values across functions GF1 to GF10. In most cases, SWRBMO approaches the theoretical optimum rapidly during the early stages of iteration, indicating strong convergence performance. On GF4, GF6 and GF8, although the best value is slightly lower than that of the IWKGJO algorithm, the SWRBMO curve remains almost flat after convergence, with minimal fluctuations. This suggests that SWRBMO is more reliable in maintaining the optimal solution.

Overall, the SWRBMO algorithm performs well in both convergence speed and stability, highlighting its strong capability in solving complex optimization problems.

#### 4.4.2. Performance Analysis Using the CEC2021 Test Function Suite

As shown in Table 7, the SWRBMO algorithm exhibits significant advantages in optimization performance. Notably, the theoretical global optimal value of the functions in the CEC2021 test function suite is 0. Specifically, on functions GF1, GF4, and GF8, SWRBMO achieves the best results across all three metrics (best value, mean value, and standard deviation), reflecting its robust overall optimization capability. On functions GF2, GF3, and GF10, SWRBMO also obtains the best optimal value and mean value, further confirming its excellent global search ability. However, on GF5, the average value and standard deviation are respectively lower than those of IWKGJO and POA. On GF6, the mean value and standard deviation of SWRBMO are inferior to those of IWKGJO. On GF7, its mean value and standard deviation are lower than those of POA. On GF9, its average value and standard deviation are respectively lower than those of POA and EOSMICOA. This indicates a slight performance gap for SWRBMO in some specific scenarios.

Overall, SWRBMO demonstrates superior performance across most functions in all three key metrics. By integrating strong global exploration with stable convergence, the algorithm presents an effective and promising solution for addressing complex optimization problems.

**Figure 5:**
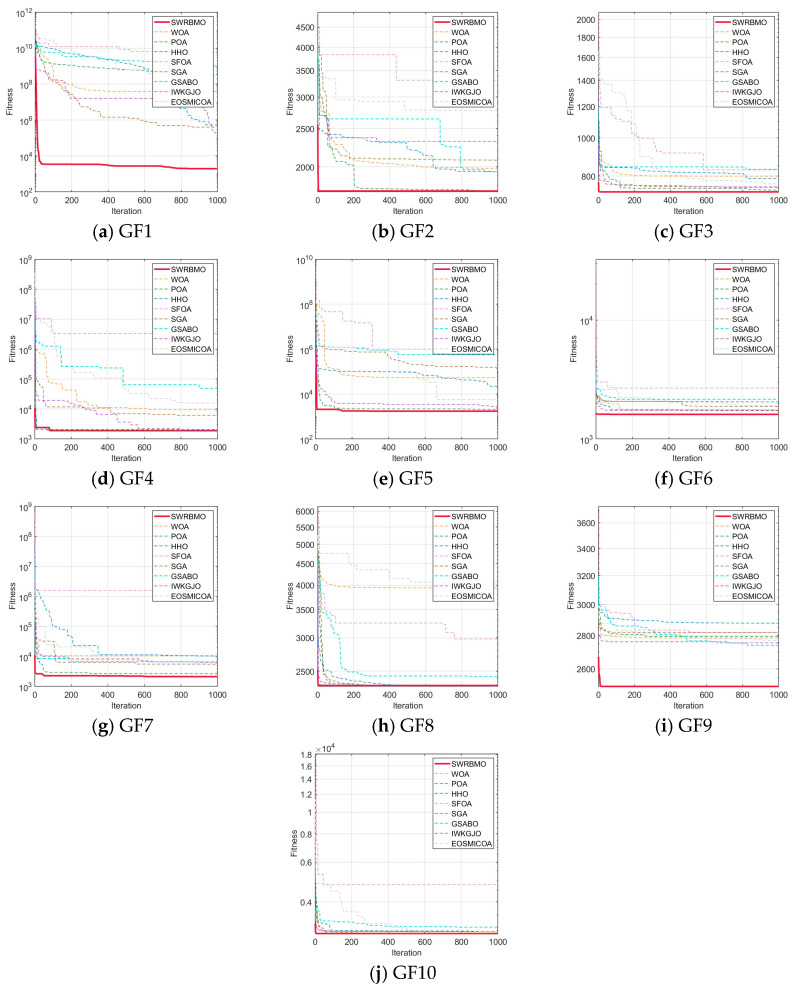
Curve comparison with other optimization algorithms on CEC2021.

As illustrated by the convergence curves in Figure 5, the SWRBMO algorithm demonstrates a markedly rapid reduction in fitness values during the initial adaptation phase across all GF1–GF10 functions, reaching low-fitness regions earlier than the comparative algorithms. This behavior reflects its significantly accelerated convergence toward the theoretical optimum. In particular, functions GF4, GF5, and GF7 exhibit a characteristic inflection point, where a secondary decline in fitness values follows an initial stagnation. Such a pattern highlights SWRBMO’s capacity to overcome premature convergence by reinitiating exploration in regions susceptible to local optima.

### 4.5. Wilcoxon Rank-Sum Test

When evaluating algorithmic performance, relying solely on indicators such as the best value, mean, and standard deviation provides an incomplete assessment. To strengthen the statistical validity of the comparisons, this study further employs the Wilcoxon rank-sum test on the CEC2005, CEC2019, and CEC2021 benchmark function suites against other optimization algorithms. In this analysis, the *p*-value measures the statistical significance of performance differences. A *p*-value below 0.05 indicates that SWRBMO achieves a statistically significant improvement over the compared algorithms, whereas a *p*-value greater than 0.05 implies no significant difference. For clarity of presentation, “+” indicates that SWRBMO outperforms the compared algorithm, “=” indicates comparable performance, “−” indicates inferior performance, and “NaN” indicates that no statistically significant difference can be established from the dataset.

#### 4.5.1. Wilcoxon Rank-Sum Test on CEC2005

Table 8 reports the results of the Wilcoxon rank-sum test on the CEC2005 benchmark functions (F1–F15), which evaluate the statistical significance of performance differences between SWRBMO and the comparison algorithms. For most functions, the *p*-values fall below the 5% significance level, with those for F2, F4–F6, F8, F12, and F13 approaching zero, indicating a highly significant advantage of SWRBMO. Conversely, the relatively larger *p*-values for F9, F10, and F11 suggest that the observed performance differences on these functions are not statistically significant. Overall, these results demonstrate that SWRBMO achieves a statistically superior optimization performance on the majority of CEC2005 functions.

#### 4.5.2. Wilcoxon Rank-Sum Test on CEC2019

The results in Table 9 show that, for the CEC2019 test suite, most *p*-values obtained from the Wilcoxon rank-sum test are below 5%, indicating significant performance differences between SWRBMO and the compared algorithms. In particular, for functions GF2, GF3, GF5, GF6, GF8, and GF10, the *p*-values are nearly zero, demonstrating a highly significant advantage of SWRBMO. It is worth noting that only on GF1 does the relatively higher *p*-value suggest no significant difference, whereas on all other functions, SWRBMO consistently achieves statistically significant improvements. Overall, these results highlight the statistical superiority of SWRBMO and its strong potential for solving complex optimization problems.

#### 4.5.3. Wilcoxon Rank-Sum Test on CEC2021

As shown in Table 10, within the CEC2021 test function suite, the SWRBMO algorithm exhibits distinct performance characteristics compared to other algorithms. Specifically, when compared with the SFOA and SGA algorithms, in GF1-GF10, SWRBMO shows significant statistical differences, highlighting its robust consistency and competitive advantages. In contrast, algorithms such as WOA, POA, HHO, GSABO and EOSMICOA demonstrate statistical significance of the performance differences across most test functions. It is worth noting that when compared with the high-performing IWKGJO algorithm, SWRBMO shows smaller performance differences on functions F1, F2, F3, and F8, with the two algorithms displaying more comparable performance.

## 5. Excellent Engineering Applications Based on SWRBMO

In the field of mechanical optimization, various engineering problems are closely intertwined with mathematical modeling. As highlighted in references [35,36], the construction of an effective optimization model primarily involves the identification of design variables, the definition of the objective function, and the systematic formulation of constraint conditions.

To validate the effectiveness and applicability of the proposed multi-strategy enhanced Red-billed Magpie Optimization algorithm (SWRBMO) in solving engineering design problems, four representative case studies are selected: the robot gripper problem, industrial refrigeration system problem, reinforced concrete beam design problem, and step cone pulley problem. Each problem is mathematically modeled, with constraint handling implemented via a penalty function approach, allowing for rigorous evaluation of the algorithm’s optimization capabilities.

The performance of SWRBMO is benchmarked against six well-known optimization algorithms: Whale Optimization Algorithm (WOA) [13], Harris Hawks Optimization (HHO) [10], Subtraction-Average-Based Optimizer (SABO) [37], Osprey Optimization Algorithm (OOA) [38], Reindeer-Inspired Metaheuristic (RIME) [39], and Red-billed Blue Magpie Optimizer (RBMO). All algorithms are executed under identical conditions, with maximum iterations *T* = 1000 and a population size *N* = 30.

### 5.1. Robot Gripper Problem

The optimization of robotic grippers poses a complex problem in the field of mechanical engineering. The main goal is to reduce the force disparity—specifically, the gap between the maximum and minimum forces—within a defined displacement interval at the gripper’s endpoint. The design process incorporates seven continuous decision variables and must comply with seven nonlinear constraints. The mathematical representation of this problem is given below:

$$x=\left(x_{1},x_{2},x_{3}{,x}_{4},x_{5},x_{6},x_{7}\right)=\left(a,b,c,d,e,f,p\right)$$

Minimize

$$f\left(x\right)=-{min}_{z}F_{k}\left(x,z\right)+{max}_{z}F_{k}\left(x,z\right)$$

Constraint condition:

$$g_{1}\left(x\right)=-Y_{min}+y\left((x),Z_{max}\right)\leq 0$$

$$g_{2}(x)=-y\left((x),Z_{max}\right)\leq 0$$

$$g_{3}(x)=Y_{max}-y\left((x),0\right)\leq 0$$

$$g_{4}(x)=y\left((x),0\right)-Y_{G}\leq 0$$

$$g_{5}(x)=l^{2}+d^{2}-{\left(a+b\right)}^{2}\leq 0$$

$$g_{6}(x)=b^{2}-{\left(a-d\right)}^{2}-{\left(l-Z_{max}\right)}^{2}\leq 0$$

$$g_{7}(x)=Z_{max}-l\leq 0$$

where

$$\alpha ={cos}^{-1}\left(\frac{a^{2}+g^{2}-b^{2}}{2ag}\right)+\phi ,\;g=\sqrt{e^{2}+{\left(z-l\right)}^{2}},$$

$$\beta ={cos}^{-1}\left(\frac{b^{2}+g^{2}-a^{2}}{2bg}\right)-\phi ,\;\phi ={tan}^{-1}\left(\frac{e}{l-z}\right)$$

$$y\left(x,z\right)=2\left(l+d+csin\left(\beta +p\right)\right)$$

$$F_{k}=\frac{Pbsin\left(\alpha +\beta \right)}{2ccos\left(\alpha \right)},\;Y_{min}=50,\;Y_{max}=100,\;Y_{G}=150,\;Z_{max}=100,\;P=100$$

Variable interval:

0≤d≤50,100≤c≤200,10≤e,a,b≤150,1≤p≤3.14,100≤f≤300

**Figure 6:**
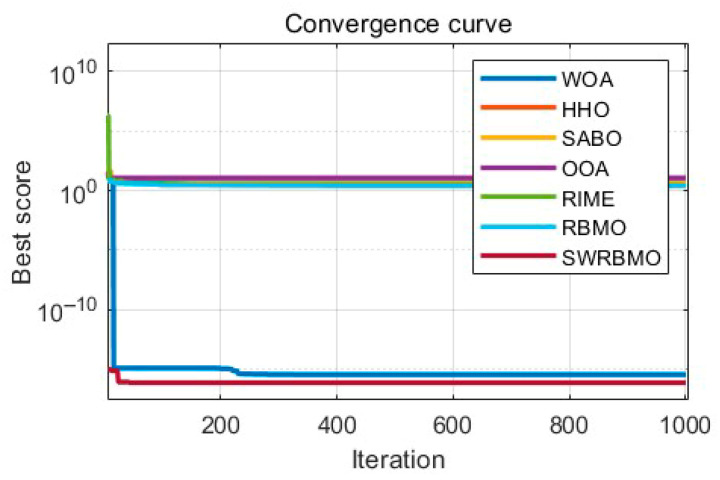
Convergence curve for robot gripper problem.

As shown in Table 11, SWRBMO achieves the best performance with the smallest deviation, yielding a result of 7.27 × 10^−17^. Compared to the HHO, SABO, OOA, RIME, and RBMO algorithms, its performance is optimized by nearly 99%. Even when benchmarked against the top-performing WOA, SWRBMO still realizes an optimization improvement of approximately 78.8%. Figure 6 illustrates that SWRBMO exhibits faster and more stable convergence during the early iterations, enabling more efficient approximation of the optimal design. This enhancement in the overall performance of SWRBMO indicates that the SWRBMO algorithm possesses significant advantages in addressing the robot gripper optimization problem.

### 5.2. Industrial Refrigeration System Problem

The problem aims to minimize the total annual cost (TAC) of the system, considering capital investment and operational expenses. This is a complex nonlinear programming problem involving both continuous and discrete decision variables. The system consists of multiple refrigeration stages and involves decision-making in areas such as compressor capacity, heat exchanger sizing, and refrigerant flow distribution. The mathematical model incorporates thermodynamic performance constraints, equipment selection rules, and energy balance equations.

Minimize

$$f\left(\bar{x}\right)=63098.88x_{2}x_{4}x_{12}+5441.5x_{2}^{2}x_{12}+115055.5x_{2}^{1.664}x_{6}\;+6172.27x_{2}^{2}x_{6}^{2}+63098.88x_{1}x_{3}x_{11}+5441.5x_{1}^{2}x_{11}\;+115055.5x_{1}^{1.664}x_{7}+6172.27x_{1}^{2}x_{5}+140.53x_{1}x_{11}\;+281.29x_{1}x_{11}+70.26x_{1}^{2}+281.29x_{1}x_{3}+281.29x_{3}^{2}\;\;\;\;\;+14437x_{8}^{1.881}x_{12}^{0.3424}x_{10}x_{14}^{-1}x_{1}^{2}x_{7}x_{9}^{-1}+20470.2x_{7}^{2.893}x_{11}^{0.316}x_{1}^{2}$$

Subject to

$$g_{1}\left(\bar{x}\right)=1.524x_{7}^{-1}\leq 1$$

$$g_{2}\left(\bar{x}\right)=1.524x_{8}^{-1}\leq 1$$

$$g_{3}\left(\bar{x}\right)=0.07789x_{1}-2x_{7}^{-1}x_{9}-1\leq 0$$

$$g_{4}\left(\bar{x}\right)=7.05305x_{9}^{-1}x_{1}^{2}x_{10}x_{8}^{-1}x_{2}^{-1}x_{14}^{-1}-1\leq 0$$

$$g_{5}\left(\bar{x}\right)=0.0833x_{13}^{-1}x_{14}-1\leq 0$$

$$g_{6}\left(\bar{x}\right)=47.136x_{2}^{0.333}x_{10}^{-1}x_{12}+62.08x_{13}^{2.1195}x_{12}^{-1}x_{8}^{0.2}x_{10}^{-1}-1.333x_{8}x_{13}^{2.1195}-1\leq 0$$

$$g_{7}\left(\bar{x}\right)=0.04771x_{10}x_{8}^{1.8812}x_{12}^{0.3424}-1\leq 0$$

$$g_{8}\left(\bar{x}\right)=0.0488{x_{9}x}_{7}^{1.893}x_{11}^{0.316}-1\leq 0$$

$$g_{9}\left(\bar{x}\right)=0.0099x_{1}x_{3}^{-1}-1\leq 0$$

$$g_{10}\left(\bar{x}\right)=0.0193x_{2}x_{4}^{-1}-1\leq 0$$

$$g_{11}\left(\bar{x}\right)=0.0298x_{1}x_{5}^{-1}-1\leq 0$$

$$g_{12}\left(\bar{x}\right)=0.056x_{2}x_{6}^{-1}-1\leq 0$$

$$g_{13}\left(\bar{x}\right)=2x_{9}^{-1}-1\leq 0$$

$$g_{14}\left(\bar{x}\right)=2x_{10}^{-1}-1\leq 0$$

$$g_{15}\left(\bar{x}\right)=x_{12}x_{11}^{-1}-1\leq 0$$

With bounds

$$0.001\leq x_{i}\leq 5\;i=1,\ldots ,14$$

**Figure 7:**
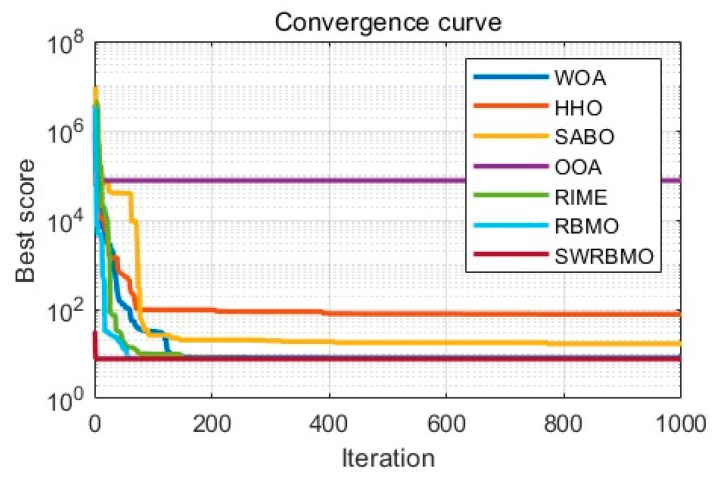
Convergence curve for industrial refrigeration system problem.

Table 12 results clearly show that the SWRBMO algorithm outperforms the other six compared algorithms. In this optimization result, the overall average performance of the SWRBMO algorithm is approximately 38.4% higher than that of the other algorithms. Combined with Figure 7, the SWRBMO algorithm demonstrates a faster and more stable convergence rate. These characteristics indicate that the SWRBMO algorithm has strong practicality in optimization problems such as industrial refrigeration system design, providing an efficient and precise solution for solving such complex optimization scenarios.

### 5.3. Reinforced Concrete Beam Design Problem

The design problem of reinforced concrete beams aims to minimize the total structural cost while ensuring structural safety and adhering to design standards. To achieve the lowest total structural cost, the area of reinforcement $\left(A_{S}=x_{1}\right)$, beam width $\left(b=x_{2}\right)$, and beam height $\left(h=x_{3}\right)$ need to be determined, with the height-to-width ratio of the beam restricted to no more than 4. This optimization problem is subject to several nonlinear constraints. Given the nonlinear characteristics of reinforced concrete and the multi-constraint nature of the problem itself, it can serve as a practical and challenging benchmark test problem for evaluating the performance of heuristic optimization algorithms.

Minimize

$$f\left(x\right)=2.9x_{1}+0.6x_{2}x_{3}$$

Subject to

$$g_{1}\left(x\right)=\frac{x_{2}}{x_{3}}-4\leq 0$$

$$g_{2}\left(x\right)=180+7.375\frac{x_{1}^{2}}{x_{3}}-x_{1}x_{2}\leq 0$$

With bounds

$$0\leq x_{1},x_{2}\leq 1$$

$$5\leq x_{3}\leq 10$$

To handle inequality constraints, this paper uses a penalty function method. Specifically, the constraint violation is incorporated into the objective function. The augmented penalty function is as follows.

$$J_{aug}\left(x\right)=f\left(x\right)+\sum_{i=1}^{n}k_{i}\cdot b_{i}$$

where $k_{i}$ is the penalty coefficient, and $b_{i}$ is calculated as follows.

$$b_{i}=\left\{\begin{array}{ll} 0 & if\;x_{i}\leq 0\\ x_{i}^{2} & if\;x_{i}>0 \end{array}\right.\;i=1,2,\ldots ,n$$

**Figure 8:**
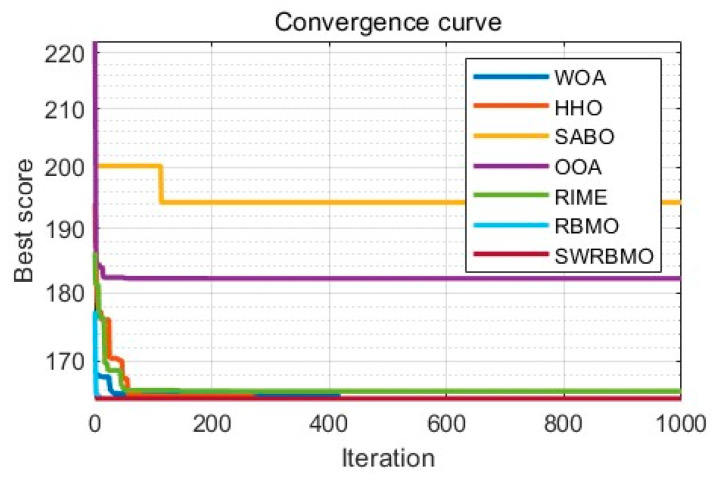
Convergence curve for reinforced concrete beam design problem.

Referring to Table 13, in the reinforced concrete beam design optimization problem, while SWRBMO’s results show subtle numerical differences from comparative algorithms (WOA, HHO, RIME, RBMO), it still has quantifiable advantages—its overall average performance is optimized by 2.4%. Though this difference seems small in isolation, it translates to material cost savings or structural performance improvements in large-scale projects (large bridges, buildings). Combined with Figure 8, SWRBMO also exhibits faster, more stable early-iteration convergence (significantly outperforming other methods), a key advantage for engineering environments with limited design time or iteration budgets.

### 5.4. Step Cone Pulley Problem

The step cone pulley optimization problem focuses on minimizing the weight of a four-step cone pulley by adjusting five design variables. Among these variables, one defines the pulley’s width, while the remaining four represent the diameters of each individual step. The optimization process is subject to eight nonlinear constraints and three linear constraints, which collectively define the feasible range for the design variables. The mathematical formulation of this problem is presented below.

Minimize

$$f(x)=\rho \omega \left[d_{1}^{2}\left\{11+{\left(\frac{N_{1}}{N}\right)}^{2}\right\}+d_{2}^{2}\left\{1+{\left(\frac{N_{2}}{N}\right)}^{2}\right\}+d_{3}^{2}\left\{1+{\left(\frac{N_{3}}{N}\right)}^{2}\right\}+d_{4}^{2}\left\{1+{\left(\frac{N_{4}}{N}\right)}^{2}\right\}\right.$$

$$h_{1}(x)=C_{1}-C_{2}=0,$$

$$h_{2}(x)=C_{1}-C_{3}=0,$$

$$h_{3}(x)=C_{1}-C_{4}=0$$

$$g_{i=1,2,3.4}\left(x\right)=-R_{i}\leq 2$$

$$g_{i=1,2,3.4}\left(\bar{x}\right)=\left(0.75\times 745.6998\right)-P_{i}\leq 0$$

where

$$C_{i}=\frac{\pi d_{i}}{2}\left(1+\frac{N_{i}}{N}\right)+\frac{{\left(\frac{N_{i}}{N}-1\right)}^{2}}{4a}+2a,i=\left(1,2,3,4\right)$$

$$R_{i}=exp\left(\mu \left\{\pi -2sin^{-1}\left\{\left(\frac{N_{i}}{N}-1\right)\frac{d_{i}}{2a}\right\}\right\}\right),i=\left(1,2,3,4\right)$$

$$P_{i}=st\omega \left(1-R_{i}\right)\frac{\pi d_{i}N_{i}}{60},i=\left(1,2,3,4\right),$$

Constant values:

$$s=1.75\;MPa,\;\mu =0.35,\;\rho =7200\;kg/m^{3},\;a=3\;mm$$

**Figure 9:**
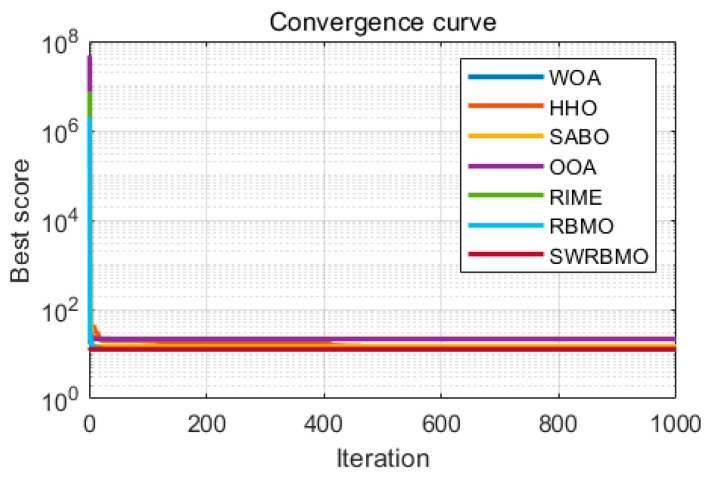
Convergence curve for step cone pulley problem.

Table 14 reveals that the SWRBMO algorithm achieves the minimum weight for the stepped cone pulley, yielding a result of 17.1. This value is far lower than those of other algorithms. Specifically, when compared with WOA, HHO, SABO, OOA, RIME and RBMO, the numerical results of the SWRBMO algorithm are on average 85 orders of magnitude lower than those of these algorithms, and the improvement is close to the theoretical limit (100%). As depicted in Figure 9, the SWRBMO algorithm consistently outperforms the other methods in both convergence accuracy and speed. This demonstrates that the SWRBMO algorithm possesses substantial potential and value for practical applications, especially in the field of mechanical engineering.

## 6. Conclusions

This study presents a multi-strategy improved Red-billed Blue Magpie Optimization Algorithm (SWRBMO), aiming to improve its effectiveness and adaptability in solving complex optimization problems. The algorithm incorporates three strategies: an adaptive T-distribution-based sinh–cosh search strategy to accelerate the convergence speed, a neighborhood-guided reinforcement strategy to help avoid local optima, and a crossover strategy to enhance population diversity and accuracy.

To evaluate the performance of the SWRBMO algorithm, a series of comprehensive experiments were conducted. The effectiveness of individual strategies was first validated through experiments on 15 standard benchmark functions. Ablation studies were performed, which not only provided further insight into the effectiveness of each strategy but also examined the synergistic effects of combining the strategies, emphasizing how they complement and enhance one another within the algorithm framework. Following this, the improved SWRBMO algorithm was tested using the CEC2019 and CEC2021 test suites. The algorithm’s performance was compared with eight representative algorithms. In addition to comparing the commonly used swarm intelligence optimization algorithms such as WHO, POA, and HHO, this study also incorporates comparisons with relatively new algorithms from recent research, namely SFOA and SGA, as well as the excellent improved algorithms, GSABO, IWKGJO, and EOSMICOA. The results show that SWRBMO achieves better accuracy and convergence on most test functions. Furthermore, the results of all three test suites (CEC2005, CEC2019, and CEC2021) were subjected to the Wilcoxon rank-sum test. It was confirmed that in most cases, SWRBMO exhibited significant statistical advantages over the compared algorithms, providing reliable empirical evidence for its outstanding optimization capabilities.

To further verify its practical applicability, SWRBMO was applied to four real-world engineering optimization problems: the robot gripper problem, the industrial refrigeration system problem, the reinforced concrete beam design problem, and the step cone pulley problem. Although the optimal objective values obtained by SWRBMO are sometimes similar to those achieved by other algorithms, its main advantages lie in faster convergence, greater solution diversity, and higher accuracy. Nevertheless, the application of SWRBMO to more complex scenarios still requires further investigation. Future research will aim to address these limitations by exploring engineering problems with higher complexity, dimensionality, constraints, or dynamic characteristics, and by extending the algorithm’s validated performance advantages to challenging fields such as wind power generation, intelligent construction, and disaster prevention.

## Figures and Tables

**Table 1:** Comparison of SWRBMO with other algorithms.

Algorithm	Inspiration Source	Main Strategy/Improvement	Strengths	Limitations
DE	Natural selection and genetic evolution mechanisms	Differential mutation and recombination	Strong global exploration via differential mutation	High cost of calculation, parameter sensitive
PSO	Group behaviors of bird flocks and fish schools	Individual and global best memory mechanism	Simple implementation, fast convergence speed	Low diversity at later stages, easy to fall into local optima, parameter sensitive
SSA	The foraging and anti-predation behaviors of sparrows	Detection and early warning mechanism	Strong global search capability	Poor convergence accuracy, easy to fall into local optima, sensitive to parameter setting
RBMO	Cooperative hunting of red-billed blue magpies	Environment-feedback-driven dynamic coordination	Strong adaptability, few parameters, good population diversity	Poor convergence accuracy; easy to fall into local optima
MDBO	The natural behavior of dung beetles, combined with improved strategies	Latin Hypercube Sampling, mean differential variation, lens imaging reverse learning and dimension-by-dimension optimization	Superior performance in terms of optimization accuracy, stability, and convergence speed	In certain complex scenarios, MDBO still faces challenges in obtaining the theoretically optimal solution
SWRBMO	Cooperative hunting of red-billed blue magpies, combined with improved strategies	Adaptive T-distribution-based sinh–cosh search strategy, neighborhood-guided reinforcement strategy and crossover strategy	Improved global search, faster convergence, higher robustness	The application of SWRBMO in certain complex scenarios requires further investigation

**Table 2:** CEC2005 benchmark function.

Function	Function Name	Dimension	Domain	Optimum Value
$F1\left(x\right)=\sum_{i=1}^{n}x_{i}^{2}$	Sphere	30/100	[−100, 100]	0
$F2\left(x\right)=\sum_{i=1}^{n}\left|x_{i}\right|+\prod_{i=1}^{n}\left|x_{i}\right|$	Schwefel’s problem 2.22	30/100	[−10, 10]	0
$F3\left(x\right)=\sum_{i=1}^{n}{\left(\sum_{j=1}^{i}x_{j}\right)}^{2}$	Schwefel’s problem 1.2	30/100	[−100, 100]	0
$F4\left(x\right)={max}_{i}\;\left|x_{i}\right|,1\leq i\leq n$	Schwefel’s problem 2.21	30/100	[−100, 100]	0
$F5\left(x\right)=\sum_{i=1}^{n-1}\left[100{\left(x_{i+1}-x_{i}^{2}\right)}^{2}+{\left(x_{i}-1\right)}^{2}\right]$	Generalized Rosenbrock function	30/100	[−30, 30]	0
$F6\left(x\right)=\sum_{i=1}^{n}{\left(\left|x_{i}+0.5\right|\right)}^{2}$	Step function	30/100	[−100, 100]	0
$F7\left(x\right)=\sum_{i=1}^{n}ix_{i}^{4}+random[0,1)$	Quartic function	30/100	[−1.28, 1.28]	0
$F8\left(x\right)=\sum_{i=1}^{n}-x_{i}sin\left(\sqrt{\left|x_{i}\right|}\right)$	Generalized Schwefel problem 2.26	30/100	[−500, 500]	−418.98
$F9\left(x\right)=\sum_{i=1}^{n}\left[x_{i}^{2}-10cos\left(2\pi x_{i}\right)+10\right]$	Generalized Rastrigin Function	30/100	[−5.12, 5.12]	0
$F10\left(x\right)=$ $-20exp\left(-0.2\sqrt{\frac{1}{n}\sum_{i=1}^{n}x_{i}^{2}}\right)-exp\left(\frac{1}{n}\sum_{i=1}^{n}cos\left(2\pi x_{i}\right)\right)+20+e$	Ackley’s function	30/100	[−32, 32]	0
$F11\left(x\right)=\frac{1}{4000}\sum_{i=1}^{n}x_{i}^{2}-\prod_{i=1}^{n}cos\left(\frac{x_{i}}{\sqrt{i}}\right)+1$	Generalized Criewank function	30/100	[−600, 600]	0
$F12\left(x\right)=$ $\frac{\pi }{n}\left\{10{sin}^{2}\left(\pi y_{i}\right)+\sum_{i=1}^{n-1}{\left(y_{i}-1\right)}^{2}\left[1+10{sin}^{2}\left(\pi y_{i+1}\right)\right]+{\left(y_{n}-1\right)}^{2}\right\}+\sum_{i=1}^{n}u\left(x_{i},10,100,4\right)$ $y_{i}=1+\frac{x_{i+1}}{4}$ $u\left(x_{i},a,k,m\right)=\left\{\begin{array}{l} k{\left(x_{i}-a\right)}^{m},x_{i}>a\\ 0,-a<x_{i}<a\\ k{\left(-x_{i}-a\right)}^{m},x_{i}<-a \end{array}\right.$	Generalized penalized function 1	30/100	[−50, 50]	0
$F_{13}\left(x\right)=0.1\left\{{sin}^{2}\left(3\pi x_{1}\right)+\sum_{i=1}^{n}{\left(x_{i}-1\right)}^{2}\left[1+{sin}^{2}\left(3\pi x_{1}+1\right)\right]\right.$ $\left.+{\left(x_{n}-1\right)}^{2}\left[1+{sin}^{2}\left(2\pi x_{n}\right)\right]\right\}+\sum_{i=1}^{n}u\left(x_{i},5,100,4\right)$	Generalized penalized function 2	30/100	[−50, 50]	0
$F14\left(x\right)={\left(\frac{1}{500}+\sum_{j=1}^{25}\frac{1}{j+\sum_{i=1}^{25}{\left(x_{i}-a_{ij}\right)}^{6}}\right)}^{-1}$	Shekell’s foxhole function	2	[−65, 65]	0
$F15\left(x\right)=\sum_{i=1}^{11}{\left[a_{i}-\frac{x_{1}\left(x_{1}^{2}+b_{1}x_{2}\right)}{b_{1}^{2}+b_{1}x_{3}+x_{4}}\right]}^{2}$	Kowalik’s function	4	[−5, 5]	0.1484

**Table 3:** Results of the CEC2005 benchmark suite for 30 dimensions and 100 dimensions.

Function	Algorithm	D = 30			D = 100		
		Best	Mean	Std	Best	Mean	Std
**F1**	SWRBMO	**0**	**0**	**0**	**0**	**0**	**0**
	RBMO1	**0**	**0**	**0**	**0**	**0**	**0**
	RBMO2	**0**	**0**	**0**	**0**	**0**	**0**
	RBMO3	**0**	**0**	**0**	**0**	**0**	**0**
	RBMO	2.119 × 10^−12^	4.328 × 10^−10^	8.708 × 10^−10^	7.837	8.600 × 10	6.821 × 10
F2	SWRBMO	**0**	**0**	**0**	**0**	**0**	**0**
	RBMO1	**0**	**0**	**0**	**0**	**0**	**0**
	RBMO2	**0**	**0**	**0**	**0**	**0**	**0**
	RBMO3	**0**	**0**	**0**	**0**	**0**	**0**
	RBMO	2.678 × 10^−7^	5.218 × 10^−6^	5.701 × 10^−6^	1.683	7.587	6.355
F3	SWRBMO	**0**	**0**	**0**	**0**	**0**	**0**
	RBMO1	**0**	**0**	**0**	**0**	**0**	**0**
	RBMO2	**0**	**0**	**0**	**0**	**0**	**0**
	RBMO3	**0**	**0**	**0**	4.860 × 10	2.636 × 10^2^	1.546 × 10^2^
	RBMO	1.481	6.354	4.948	4.392 × 10^3^	9.835 × 10^3^	3.580 × 10^3^
F4	SWRBMO	**0**	**0**	**0**	**0**	**0**	**0**
	RBMO1	**0**	**0**	**0**	**0**	**0**	**0**
	RBMO2	**0**	**0**	**0**	**0**	**0**	**0**
	RBMO3	**0**	**0**	**0**	2.483 × 10^−5^	1.367 × 10^−4^	9.041 × 10^−5^
	RBMO	1.978 × 10^−1^	7.501 × 10^−1^	4.057 × 10^−1^	9.980	1.417 × 10	1.883
F5	SWRBMO	1.736 × 10	**1.841 × 10**	4.375 × 10^−1^	9.187 × 10	**9.233 × 10**	**1.965 × 10^−1^**
	RBMO1	2.216 × 10	2.326 × 10	7.388 × 10^−1^	9.352 × 10	9.476 × 10	6.550 × 10^−1^
	RBMO2	2.581 × 10	2.649 × 10	**3.579 × 10^−1^**	9.651 × 10	9.746 × 10	6.132 × 10^−1^
	RBMO3	**6.599 × 10^−5^**	9.146	1.198 × 10	**3.724 × 10^−2^**	1.129 × 10^2^	7.385 × 10
	RBMO	2.003 × 10	4.240 × 10	3.510 × 10	1.542 × 10^3^	4.811 × 10^3^	2.890 × 10^3^
F6	SWRBMO	**0**	**0**	**0**	**0**	**0**	**0**
	RBMO1	6.968 × 10^−10^	4.120 × 10^−8^	1.136 × 10^−7^	5.016 × 10^−2^	2.825 × 10^−1^	2.154 × 10^−1^
	RBMO2	3.690 × 10^−5^	3.019 × 10^−2^	1.136 × 10^−1^	3.810	5.587	1.119
	RBMO3	**0**	**0**	**0**	**0**	**0**	**0**
	RBMO	1.825 × 10^−12^	1.092 × 10^−9^	2.374 × 10^−9^	2.022 × 10	7.623 × 10	8.510 × 10
F7	SWRBMO	1.509 × 10^−4^	1.320 × 10^−3^	1.170 × 10^−3^	1.208 × 10^−5^	1.325 × 10^−3^	1.282 × 10^−3^
	RBMO1	**9.051 × 10^−6^**	**5.423 × 10^−5^**	**5.658 × 10^−5^**	**9.934 × 10^−7^**	**5.295 × 10^−5^**	**4.426 × 10^−5^**
	RBMO2	2.442 × 10^−5^	3.520× 10^−5^	3.409 × 10^−4^	1.799 × 10^−5^	2.474 × 10^−4^	2.158 × 10^−4^
	RBMO3	1.008 × 10^−2^	2.160 × 10^−2^	7.055 × 10^−3^	1.084 × 10^−1^	1.885 × 10^−1^	4.892 × 10^−2^
	RBMO	3.384 × 10^−3^	1.188 × 10^−2^	7.101 × 10^−3^	1.565 × 10^−1^	4.141 × 10^−1^	2.360 × 10^−1^
F8	SWRBMO	**−1.257 × 10^4^**	**−1.257 × 10^4^**	5.394 × 10^−12^	**−4.190 × 10^4^**	**−4.190 × 10^4^**	2.879 × 10^−11^
	RBMO1	−1.209 × 10^4^	−9.857 × 10^3^	1.060 × 10^3^	−3.378 × 10^4^	−2.775 × 10^4^	2.899 × 10^3^
	RBMO2	−8.578 × 10^3^	−7.483 × 10^3^	6.211 × 10^2^	−2.641 × 10^4^	−2.172 × 10^4^	2.126 × 10^3^
	RBMO3	**−1.257 × 10^4^**	**−1.257 × 10^4^**	**3.411 × 10^−12^**	**−4.190 × 10^4^**	**−4.190 × 10^4^**	**2.027 × 10^−11^**
	RBMO	−9.888 × 10^3^	−9.023 × 10^3^	6.979 × 10^2^	−2.902 × 10^4^	−2.517 × 10^4^	2.502 × 10^3^
F9	SWRBMO	**0**	**0**	**0**	**0**	**0**	**0**
	RBMO1	**0**	**0**	**0**	**0**	**0**	**0**
	RBMO2	**0**	**0**	**0**	**0**	**0**	**0**
	RBMO3	**0**	2.463 × 10^−14^	3.859 × 10^−14^	1.137 × 10^−12^	1.709 × 10^−12^	3.589 × 10^−13^
	RBMO	2.215 × 10	4.560 × 10	1.596 × 10	1.352 × 10^2^	3.060 × 10^2^	5.919 × 10
F10	SWRBMO	**4.441 × 10^−16^**	**4.441 × 10^−16^**	**0**	**4.441 × 10^−16^**	**4.441 × 10^−16^**	**0**
	RBMO1	**4.441 × 10^−16^**	**4.441 × 10^−16^**	**0**	**4.441 × 10^−16^**	**4.441 × 10^−16^**	**0**
	RBMO2	**4.441 × 10^−16^**	**4.441 × 10^−16^**	**0**	**4.441 × 10^−16^**	**4.441 × 10^−16^**	**0**
	RBMO3	7.550 × 10^−15^	1.039 × 10^−14^	5.144 × 10^−15^	9.281 × 10^−14^	1.351 × 10^−13^	1.622 × 10^−14^
	RBMO	8.881 × 10^−7^	3.420 × 10^−1^	5.848 × 10^−1^	2.823	5.086	1.041
F11	SWRBMO	**0**	**0**	**0**	**0**	**0**	**0**
	RBMO1	**0**	**0**	**0**	**0**	**0**	**0**
	RBMO2	**0**	**0**	**0**	**0**	**0**	**0**
	RBMO3	**0**	1.780 × 10^−2^	4.550 × 10^−2^	**0**	**0**	**0**
	RBMO	6.242 × 10^−12^	1.304 × 10^−2^	2.088 × 10^−2^	1.099	1.634	4.901 × 10^−1^
F12	SWRBMO	**1.571 × 10^−32^**	**1.571 × 10^−32^**	**5.567 × 10^−48^**	**4.712 × 10^−33^**	**4.712 × 10^−33^**	**1.392 × 10^−48^**
	RBMO1	2.247 × 10^−11^	4.160 × 10^−9^	1.750 × 10^−8^	4.298 × 10^−4^	2.820 × 10^−3^	1.965 × 10^−3^
	RBMO2	1.283 × 10^−6^	5.491 × 10^−3^	1.914 × 10^−2^	3.542 × 10^−2^	7.365 × 10^−2^	2.658 × 10^−2^
	RBMO3	**1.571 × 10^−32^**	**1.571 × 10^−32^**	**5.567 × 10^−48^**	**4.712 × 10^−33^**	**4.712 × 10^−33^**	**1.392 × 10^−48^**
	RBMO	3.964 × 10^−11^	1.013 × 10^−1^	2.281 × 10^−1^	2.902	7.073	3.223
F13	SWRBMO	**1.350 × 10^−32^**	**1.350 × 10^−32^**	**5.567 × 10^−48^**	**1.350 × 10^−32^**	**1.350 × 10^−32^**	**5.567 × 10^−48^**
	RBMO1	1.399 × 10^−9^	2.583 × 10^−2^	3.680 × 10^−2^	1.220	5.025	3.704
	RBMO2	1.093 × 10^−3^	5.568 × 10^−1^	7.302 × 10^−1^	5.298	9.506	1.101
	RBMO3	**1.350 × 10^−32^**	**1.350 × 10^−32^**	**5.567 × 10^−48^**	**1.350 × 10^−32^**	**1.350 × 10^−32^**	**5.567 × 10^−48^**
	RBMO	7.926 × 10^−12^	4.395 × 10^−3^	5.475 × 10^−3^	6.060 × 10	1.103 × 10^2^	3.142 × 10

**Table 4:** Results of the CEC2005 benchmark suite for fixed dimensions.

Function	Algorithm	Best	Mean	Std	Function	Best	Mean	Std
F14	SWRBMO	**9.980 × 10^−1^**	**9.980 × 10^−1^**	**0**	F15	**3.075 × 10^−4^**	**3.075 × 10^−4^**	**8.860 × 10^−15^**
	RBMO1	**9.980 × 10^−1^**	**9.980 × 10^−1^**	**0**		**3.075 × 10^−4^**	4.380 × 10^−3^	8.276 × 10^−3^
	RBMO2	**9.980 × 10^−1^**	1.064	2.567 × 10^−1^		**3.075 × 10^−4^**	3.251 × 10^−3^	6.963 × 10^−3^
	RBMO3	**9.980 × 10^−1^**	1.392	1.525		**3.075 × 10^−4^**	4.296 × 10^−4^	3.222 × 10^−4^
	RBMO	**9.980 × 10^−1^**	**9.980 × 10^−1^**	**0**		**3.075 × 10^−4^**	4.502 × 10^−3^	8.217 × 10^−3^

**Table 5:** Performance comparison in ablation study.

Function	Algorithm	Best	Mean	Std	Function	Best	Mean	Std
F1	SRWRBO	**0**	**0**	**0**	F7	1.509 × 10^−4^	1.320 × 10^−3^	1.170 × 10^−3^
	BRBMO	**0**	**0**	**0**		**8.520 × 10^−6^**	**7.360 × 10^−5^**	**4.970 × 10^−5^**
	MRBMO	**0**	**0**	**0**		1.600 × 10^−4^	8.320 × 10^−4^	7.270 × 10^−4^
	NRBMO	**0**	**0**	**0**		1.760 × 10^−5^	2.270 × 10^−4^	2.640 × 10^−4^
	RBMO	2.119 × 10^−12^	4.328 × 10^−10^	8.708 × 10^−10^		3.384 × 10^−3^	1.188 × 10^−2^	7.101 × 10^−3^
F2	SRWRBO	**0**	**0**	**0**	F8	−1.257 × 10^4^	−1.257 × 10^4^	**−5.394 × 10^−12^**
	BRBMO	**0**	**0**	**0**		−1.100 × 10^4^	−1.000 × 10^4^	8.208 × 10^2^
	MRBMO	**0**	**0**	**0**		**−1.260 × 10^4^**	**−1.260 × 10^4^**	3.330 × 10^−12^
	NRBMO	**0**	**0**	**0**		**−1.260 × 10^4^**	**−1.260 × 10^4^**	4.670 × 10^−12^
	RBMO	2.678 × 10^−7^	5.218 × 10^−6^	5.701 × 10^−6^		−9.888 × 10^3^	−9.023 × 10^3^	6.979 × 10^2^
F3	SRWRBMO	**0**	**0**	**0**	F9	**0**	**0**	**0**
	BRBMO	**0**	**0**	**0**		**0**	**0**	**0**
	MRBMO	**0**	**0**	**0**		**0**	**0**	**0**
	NRBMO	**0**	**0**	**0**		**0**	**0**	**0**
	RBMO	1.481	6.354	4.948		2.215 × 10	4.560 × 10	1.596 × 10
F4	SRWRBMO	**0**	**0**	**0**	F10	**4.441 × 10^−16^**	**4.441 × 10^−16^**	**0**
	BRBMO	**0**	**0**	**0**		**4.441 × 10^−16^**	**4.441 × 10^−16^**	**0**
	MRBMO	**0**	**0**	**0**		**4.441 × 10^−16^**	**4.441 × 10^−16^**	**0**
	NRBMO	**0**	**0**	**0**		**4.441 × 10^−16^**	**4.441 × 10^−16^**	**0**
	RBMO	1.978 × 10^−1^	7.501 × 10^−1^	4.057 × 10^−1^		8.881 × 10^−7^	3.420 × 10^−1^	5.848 × 10^−1^
F5	SRWRBMO	1.736 × 10	1.841 × 10	4.375 × 10^−1^	F11	**0**	**0**	**0**
	BRBMO	2.142 × 10	2.312 × 10	5.252 × 10^−1^		**0**	**0**	**0**
	MRBMO	1.717 × 10	1.828 × 10	5.492 × 10^−1^		**0**	**0**	**0**
	NRBMO	**4.930 × 10^−17^**	**2.242 × 10^−3^**	**5.995 × 10^−3^**		**0**	**0**	**0**
	RBMO	2.003 × 10	4.240 × 10	3.510 × 10		6.242 × 10^−12^	1.304 × 10^−2^	2.088 × 10^−2^
F6	SRWRBMO	**0**	**0**	**0**	F12	**1.571 × 10^−32^**	**1.571 × 10^−32^**	**5.567 × 10^−48^**
	BRBMO	1.360 × 10^−10^	1.860 × 10^−9^	1.820 × 10^−9^		6.761 × 10^−11^	3.961 × 10^−10^	3.638 × 10^−10^
	MRBMO	**0**	**0**	**0**		**1.571 × 10^−32^**	**1.571 × 10^−32^**	**5.567 × 10^−48^**
	NRBMO	**0**	**0**	**0**		**1.571 × 10^−32^**	**1.571 × 10^−32^**	**5.567 × 10^−48^**
	RBMO	1.825 × 10^−12^	1.092 × 10^−9^	2.374 × 10^−9^		3.964 × 10^−11^	1.013 × 10^−1^	2.281 × 10^−1^

**Table 6:** Results on CEC2019 test suite.

Function		SWRBMO	WOA	POA	HHO	SFOA	SGA	GSABO	IWKGJO	EOSMICOA
GF1	Best	**1**	3.255 × 10^4^	**1**	**1**	5.117 × 10^7^	**1**	**1**	**1**	**1**
	Mean	**1**	7.539 × 10^6^	**1**	**1**	6.441 × 10^8^	**1**	**1**	1.064	1.836 × 10^6^
	std	**0**	1.018 × 10^7^	**0**	**0**	3.856 × 10^8^	**0**	**0**	2.928 × 10^−1^	3.634 × 10^6^
GF2	Best	**4.205**	2.853 × 10^3^	4.238	4.814	4.175 × 10^3^	8.461 × 10^3^	5.000	4.246	6.090
	Mean	**4.388**	6.659 × 10^3^	4.325	4.987	2.208 × 10^4^	1.710 × 10^4^	5.000	4.541	6.543 × 10^2^
	std	1.445 × 10^−1^	2.914 × 10^3^	8.439 × 10^−2^	4.785 × 10^−2^	6.656 × 10^3^	5.936 × 10^3^	**0**	3.251 × 10^−1^	9.026 × 10^2^
GF3	Best	**1**	1.230	1.002	2.549	1.024 × 10	2.157	3.365	1.034	2.221
	Mean	**1.802**	4.289	1.979	4.741	1.147 × 10	4.205	1.070 × 10^20^	2.620	4.233
	std	1.610	2.045	9.180 × 10^−1^	1.463	**5.710 × 10^−1^**	1.445	8.680 × 10^19^	1.722	1.125
GF4	Best	1.393 × 10	2.009 × 10	1.991 × 10	2.401 × 10	4.572 × 10	1.530 × 10	6.201 × 10	**4.287**	3.333 × 10
	Mean	3.868 × 10	4.861 × 10	3.960 × 10	5.123 × 10	1.155 × 10^2^	5.096 × 10	1.009 × 10^2^	**1.984 × 10**	4.370 × 10
	std	1.550 × 10	1.650 × 10	1.268 × 10	1.726 × 10	3.309 × 10	1.605 × 10	1.385 × 10	**8.876**	7.660
GF5	Best	**1.126**	1.522	1.618	1.714	1.662 × 10	1.416	1.730 × 10	1.196	3.003
	Mean	**1.508**	2.219	6.903	1.979	1.216 × 10^2^	1.891	5.241 × 10	1.588	4.612
	std	**3.203 × 10^−1^**	5.225 × 10^−1^	1.182 × 10	2.179 × 10^−1^	5.593 × 10	3.531 × 10^−1^	2.528 × 10	3.475 × 10^−1^	2.607
GF6	Best	2.545	6.126	2.996	4.815	9.774	2.880	9.273	**1.645**	4.715
	Mean	**2.484**	9.066	4.934	7.637	1.293 × 10	7.071	1.060 × 10	2.815	6.763
	std	1.604	1.440	1.529	1.683	1.284	1.996	**6.392 × 10^−1^**	8.334 × 10^−1^	1.384
GF7	Best	**2.801 × 10**	5.316 × 10^2^	4.955 × 10^2^	7.017 × 10^2^	1.400 × 10^3^	7.762 × 10^2^	1.228 × 10^3^	2.529 × 10^2^	1.214 × 10^3^
	Mean	**7.341 × 10^2^**	1.520 × 10^3^	9.116 × 10^2^	1.063 × 10^3^	2.238 × 10^3^	1.301 × 10^3^	1.685 × 10^3^	7.828 × 10^2^	1.640 × 10^3^
	std	**2.335 × 10^2^**	4.891 × 10^2^	2.622 × 10^2^	2.373 × 10^2^	3.414 × 10^2^	2.611 × 10^2^	2.508 × 10^2^	2.383 × 10^2^	2.445 × 10^2^
GF8	Best	**2.613**	4.138	3.552	4.290	4.744	3.836	4.227	3.140	4.321
	Mean	**2.561**	4.611	4.111	4.827	5.251	4.478	4.828	3.850	4.668
	std	3.758	2.601 × 10^−1^	2.964 × 10^−1^	2.286 × 10^−1^	**2.146 × 10^−1^**	3.724 × 10^−1^	2.222 × 10^−1^	3.411 × 10^−1^	2.193 × 10^−1^
GF9	Best	**1.075**	1.166	1.087	1.121	1.671	1.080	1.303	1.080	1.187
	Mean	**1.230**	1.415	1.349	1.427	4.560	1.317	3.276	1.292	1.318
	std	9.958 × 10^−2^	2.079 × 10^−1^	5.488 × 10^−1^	2.056 × 10^−1^	9.820 × 10^−1^	1.710 × 10^−1^	9.375 × 10^−1^	**7.500 × 10^−2^**	9.260 × 10^−2^
GF10	Best	2.100 × 10	2.104 × 10	1.409 × 10	2.100 × 10	2.130 × 10	2.100 × 10	2.120 × 10	**1.630**	2.125 × 10
	Mean	2.100 × 10	2.127 × 10	2.062 × 10	2.117 × 10	2.180 × 10	2.110 × 10	2.140 × 10	**1.880 × 10**	2.149 × 10
	std	**4.062 × 10^−3^**	1.482 × 10^−1^	1.808	9.136 × 10^−2^	1.518 × 10^−1^	1.238 × 10^−1^	1.103 × 10^−1^	6.539	1.147 × 10^−1^

**Table 7:** Results on CEC2021 test function suite.

Function		SWRBMO	WOA	POA	HHO	SFOA	SGA	GSABO	IWKGJO	EOSMICOA
**GF1**	Best	**1.344 × 10^2^**	5.497 × 10^5^	3.730 × 10^4^	1.415 × 10^5^	1.860 × 10^9^	2.039 × 10^4^	9.400 × 10^8^	1.107 × 10^4^	1.335 × 10^8^
	Mean	**2.533 × 10^3^**	1.306 × 10^7^	1.220 × 10^8^	4.584 × 10^5^	1.125 × 10^10^	4.398 × 10^5^	4.231 × 10^9^	1.036 × 10^5^	3.857 × 10^8^
	std	**2.268 × 10^3^**	2.101 × 10^7^	2.120 × 10^8^	1.734 × 10^5^	5.627 × 10^9^	1.412 × 10^6^	2.213 × 10^9^	9.840 × 10^4^	2.271 × 10^8^
GF2	Best	**1.254 × 10^3^**	1.693 × 10^3^	1.280 × 10^3^	1.550 × 10^3^	2.696 × 10^3^	1.639 × 10^3^	2.016 × 10^3^	1.367 × 10^3^	2.067 × 10^3^
	Mean	**1.806 × 10^3^**	2.298 × 10^3^	1.810 × 10^3^	2.022 × 10^3^	3.305 × 10^3^	2.190 × 10^3^	2.609 × 10^3^	1.810 × 10^3^	2.599 × 10^3^
	std	2.663 × 10^2^	2.485 × 10^2^	2.240 × 10^2^	2.351 × 10^2^	1.933 × 10^2^	3.024 × 10^2^	**2.230 × 10^2^**	3.584 × 10^2^	2.259 × 10^2^
GF3	Best	**7.230 × 10^2^**	7.287 × 10^2^	7.270 × 10^2^	7.550 × 10^2^	8.393 × 10^2^	7.337 × 10^2^	7.658 × 10^2^	7.233 × 10^2^	7.484 × 10^2^
	Mean	**7.408 × 10^2^**	7.825 × 10^2^	7.620 × 10^2^	7.953 × 10^2^	1.015 × 10^3^	7.646 × 10^2^	8.231 × 10^2^	7.412 × 10^2^	7.643 × 10^2^
	std	2.230 × 10	3.136 × 10	2.060 × 10	2.099 × 10	1.467 × 10^2^	1.726 × 10	1.665 × 10	**1.026 × 10**	1.073 × 10
GF4	Best	**1.901 × 10^3^**	2.112 × 10^3^	1.910 × 10^3^	2.083 × 10^3^	1.185 × 10^4^	1.967 × 10^3^	2.567 × 10^3^	1.916 × 10^3^	7.824 × 10^3^
	Mean	**3.631 × 10^3^**	5.325 × 10^4^	1.950 × 10^3^	1.443 × 10^4^	8.702 × 10^6^	8.563 × 10^3^	1.157 × 10^4^	5.271 × 10^3^	2.030 × 10^4^
	std	**3.677 × 10^3^**	8.330 × 10^4^	3.870 × 10^3^	1.331 × 10^4^	2.232 × 10^7^	7.984 × 10^3^	1.037 × 10^4^	4.970 × 10^3^	6.796 × 10^3^
GF5	Best	**1.714 × 10^3^**	9.532 × 10^3^	2.000 × 10^3^	3.078 × 10^3^	3.940 × 10^4^	3.441 × 10^3^	1.354 × 10^5^	1.820 × 10^3^	3.029 × 10^3^
	Mean	2.373 × 10^3^	3.206 × 10^5^	2.990 × 10^3^	6.438 × 10^4^	9.657 × 10^6^	3.196 × 10^4^	7.253 × 10^5^	**2.366 × 10^3^**	3.055 × 10^4^
	std	2.123 × 10^3^	3.284 × 10^5^	**1.660 × 10^3^**	8.021 × 10^4^	1.935 × 10^7^	2.910 × 10^4^	1.995 × 10^5^	3.804 × 10^2^	3.586 × 10^4^
GF6	Best	**1.600 × 10^3^**	1.624 × 10^3^	1.600 × 10^3^	1.622 × 10^3^	1.906 × 10^3^	1.620 × 10^3^	1.764 × 10^3^	1.606 × 10^3^	1.636 × 10^3^
	Mean	1.806 × 10^3^	1.893 × 10^3^	1.770 × 10^3^	1.885 × 10^3^	2.348 × 10^3^	1.822 × 10^3^	2.078 × 10^3^	**1.724 × 10^3^**	1.844 × 10^3^
	std	1.355 × 10^2^	1.519 × 10^2^	1.250 × 10^2^	1.539 × 10^2^	2.327 × 10^2^	1.519 × 10^2^	1.071 × 10^2^	**1.302 × 10^2^**	1.100 × 10^2^
GF7	Best	2.243 × 10^3^	2.780 × 10^3^	**2.140 × 10^3^**	3.724 × 10^3^	1.054 × 10^4^	2.753 × 10^3^	6.052 × 10^3^	2.541 × 10^3^	3.011 × 10^3^
	Mean	6.928 × 10^3^	9.828 × 10^3^	**2.490 × 10^3^**	1.018 × 10^4^	8.654 × 10^5^	8.043 × 10^3^	1.466 × 10^4^	8.138 × 10^3^	8.997 × 10^3^
	std	5.581 × 10^3^	5.965 × 10^3^	**2.750 × 10^2^**	5.019 × 10^3^	1.422 × 10^6^	4.392 × 10^3^	6.762 × 10^3^	3.779 × 10^3^	3.416 × 10^3^
GF8	Best	**2.242 × 10^3^**	2.268 × 10^3^	2.300 × 10^3^	2.275 × 10^3^	2.365 × 10^3^	2.303 × 10^3^	2.386 × 10^3^	2.301 × 10^3^	2.338 × 10^3^
	Mean	**2.311 × 10^3^**	2.400 × 10^3^	2.340 × 10^3^	2.393 × 10^3^	3.326 × 10^3^	2.311 × 10^3^	2.762 × 10^3^	2.316 × 10^3^	3.867 × 10^3^
	std	**1.448 × 10**	3.264 × 10^2^	4.560 × 10	3.118 × 10^2^	6.144 × 10^2^	4.964	4.070 × 10^2^	3.100	5.415 × 10^2^
GF9	Best	**2.500 × 10^3^**	2.560 × 10^3^	2.500 × 10^3^	2.501 × 10^3^	2.804 × 10^3^	2.502 × 10^3^	2.624 × 10^3^	2.501 × 10^3^	2.760 × 10^3^
	Mean	2.760 × 10^3^	2.772 × 10^3^	**2.650 × 10^3^**	2.810 × 10^3^	2.848 × 10^3^	2.757 × 10^3^	2.849 × 10^3^	2.739 × 10^3^	2.772 × 10^3^
	std	5.093 × 10	4.602 × 10	1.330 × 10^2^	7.255 × 10	3.828 × 10	8.842 × 10	1.012 × 10^2^	5.023 × 10	**8.472**
GF10	Best	**2.898 × 10^3^**	2.691 × 10^3^	2.900 × 10^3^	2.898 × 10^3^	2.953 × 10^3^	2.899 × 10^3^	2.981 × 10^3^	2.898 × 10^3^	2.929 × 10^3^
	Mean	**2.923 × 10^3^**	2.941 × 10^3^	2.940 × 10^3^	2.935 × 10^3^	3.884 × 10^3^	2.940 × 10^3^	3.369 × 10^3^	2.930 × 10^3^	2.956 × 10^3^
	std	2.304 × 10	5.136 × 10	2.890 × 10	3.020 × 10	6.205 × 10^2^	3.317 × 10	2.457 × 10^2^	2.360 × 10	**1.854 × 10**

**Table 8:** Results of the rank-sum test on CEC2005.

Function	SWRBMO- WOA	SWRBMO- POA	SWRBMO- HHO	SWRBMO- SFOA	SWRBMO- SGA	SWRBMO- GSABO	SWRBMO-IWKGJO	SWRBMO-EOSMICOA
F1	1.212 × 10^−12^	1.212 × 10^−12^	1.212 × 10^−12^	1.212 × 10^−12^	1.212 × 10^−12^	**1**	**1**	1.212 × 10^−12^
F2	1.212 × 10^−12^	1.212 × 10^−12^	1.212 × 10^−12^	1.212 × 10^−12^	1.212 × 10^−12^	1.657 × 10^−11^	1.212 × 10^−12^	1.212 × 10^−12^
F3	1.212 × 10^−12^	1.212 × 10^−12^	1.212 × 10^−12^	1.212 × 10^−12^	1.212 × 10^−12^	**1**	1.104 × 10^−2^	1.212 × 10^−12^
F4	1.212 × 10^−12^	1.212 × 10^−12^	1.212 × 10^−12^	1.212 × 10^−12^	1.212 × 10^−12^	4.574 × 10^−12^	1.212 × 10^−12^	1.212 × 10^−12^
F5	3.020 × 10^−11^	3.020 × 10^−11^	6.066 × 10^−11^	3.020 × 10^−11^	3.020 × 10^−11^	1.329 × 10^−10^	3.020 × 10^−11^	3.020 × 10^−11^
F6	1.212 × 10^−12^	1.212 × 10^−12^	1.212 × 10^−12^	1.212 × 10^−12^	1.212 × 10^−12^	1.212 × 10^−12^	1.212 × 10^−12^	1.212 × 10^−12^
F7	**7.845 × 10^−1^**	4.311 × 10^−8^	1.429 × 10^−8^	3.020 × 10^−11^	**5.395 × 10^−1^**	1.695 × 10^−9^	4.200 × 10^−10^	6.669 × 10^−3^
F8	1.666 × 10^−11^	1.666 × 10^−11^	1.666 × 10^−11^	1.639 × 10^−11^	1.666 × 10^−11^	1.666 × 10^−11^	1.666 × 10^−11^	1.666 × 10^−11^
F9	**1**	**1**	**1**	1.212 × 10^−12^	1.212 × 10^−12^	**1**	**1**	**3.337 × 10^−1^**
F10	2.641 × 10^−5^	8.986 × 10^−11^	**1**	1.212 × 10^−12^	1.212 × 10^−12^	**1**	**1**	1.212 × 10^−12^
F11	1.104 × 10^−2^	**1**	**1**	1.212 × 10^−12^	1.212 × 10^−12^	**1**	**3.337 × 10^−1^**	1.104 × 10^−2^
F12	1.212 × 10^−12^	1.212 × 10^−12^	1.212 × 10^−12^	1.212 × 10^−12^	1.212 × 10^−12^	1.212 × 10^−12^	1.212 × 10^−12^	1.212 × 10^−12^
F13	1.212 × 10^−12^	1.212 × 10^−12^	1.212 × 10^−12^	1.212 × 10^−12^	1.212 × 10^−12^	1.212 × 10^−12^	1.212 × 10^−12^	1.212 × 10^−12^
F14	2.364 × 10^−12^	**1.607 × 10^−1^**	2.364 × 10^−12^	2.364 × 10^−12^	2.364 × 10^−12^	2.364 × 10^−12^	2.364 × 10^−12^	2.364 × 10^−12^
F15	1.212 × 10^−12^	1.212 × 10^−12^	1.212 × 10^−12^	1.212 × 10^−12^	1.212 × 10^−12^	**1**	**1**	1.212 × 10^−12^
+/=/−	13/0/2	12/0/3	12/0/3	15/0/0	14/0/1	9/0/6	10/0/5	14/0/1

**Table 9:** Results of the rank-sum test on CEC2019.

Function	SWRBMO- WOA	SWRBMO- POA	SWRBMO- HHO	SWRBMO- SFOA	SWRBMO- SGA	SWRBMO- GSABO	SWRBMO-IWKGJO	SWRBMO-EOSMICOA
GF1	1.212 × 10^−12^	**1**	**1**	1.210 × 10^−12^	**1**	**1**	4.574 × 10^−12^	1.210 × 10^−12^
GF2	3.020 × 10^−11^	5.264 × 10^−4^	1.015 × 10^−11^	3.020 × 10^−11^	3.020 × 10^−11^	1.212 × 10^−12^	2.704 × 10^−2^	3.020 × 10^−11^
GF3	3.020 × 10^−11^	5.573 × 10^−10^	5.573 × 10^−10^	3.020 × 10^−11^	8.480 × 10^−9^	3.020 × 10^−11^	8.485 × 10^−9^	5.570 × 10^−10^
GF4	1.606 × 10^−6^	1.501 × 10^−2^	1.337 × 10^−5^	3.020 × 10^−11^	3.770 × 10^−4^	8.153 × 10^−11^	**8.771 × 10^−2^**	4.860 × 10^−3^
GF5	1.698 × 10^−8^	1.558 × 10^−8^	3.352 × 10^−8^	3.020 × 10^−11^	6.770 × 10^−5^	3.020 × 10^−11^	9.069 × 10^−3^	3.020 × 10^−11^
GF6	4.183 × 10^−9^	5.943 × 10^−2^	8.146 × 10^−5^	5.490 × 10^−11^	4.080 × 10^−5^	3.020 × 10^−11^	3.831 × 10^−5^	1.250 × 10^−5^
GF7	1.558 × 10^−8^	4.856 × 10^−3^	2.034 × 10^−9^	3.020 × 10^−11^	4.310 × 10^−8^	6.066 × 10^−11^	**9.117 × 10^−1^**	3.020 × 10^−11^
GF8	8.485 × 10^−9^	6.353 × 10^−2^	2.439 × 10^−9^	3.330 × 10^−11^	3.830 × 10^−6^	1.857 × 10^−9^	4.637 × 10^−3^	4.570 × 10^−9^
GF9	1.383 × 10^−2^	8.684 × 10^−3^	4.459 × 10^−4^	3.020 × 10^−11^	**3.632 × 10^−1^**	4.975 × 10^−11^	1.250 × 10^−5^	**2.010 × 10^−1^**
GF10	3.020 × 10^−11^	9.514 × 10^−6^	6.722 × 10^−10^	3.020 × 10^−11^	2.230 × 10^−9^	3.020 × 10^−11^	1.360 × 10^−7^	3.020 × 10^−11^
+/=/−	10/0/0	9/0/1	9/0/1	10/0/0	8/0/2	9/0/1	8/0/2	9/0/1

**Table 10:** Results of the rank-sum test on CEC2021.

Function	SWRBMO- WOA	SWRBMO- POA	SWRBMO- HHO	SWRBMO- SFOA	SWRBMO- SGA	SWRBMO- GSABO	SWRBMO-IWKGJO	SWRBMO-EOSMICOA
GF1	1.212 × 10^−12^	1.210 × 10^−12^	1.210 × 10^−12^	3.311 × 10^−20^	3.311 × 10^−20^	3.371 × 10^−2^	**1**	1.212 × 10^−12^
GF2	1.089 × 10^−2^	2.142 × 10^−2^	4.550 × 10^−2^	3.311 × 10^−20^	3.311 × 10^−20^	2.158 × 10^−2^	**1**	2.853 × 10^−4^
GF3	**1**	**1**	2.620 × 10^−3^	3.311 × 10^−20^	3.311 × 10^−20^	**1**	**1**	1.212 × 10^−12^
GF4	2.158 × 10^−2^	1.104 × 10^−2^	**1**	3.311 × 10^−20^	3.311 × 10^−20^	**1**	1.370 × 10^−3^	**1.608 × 10^−1^**
GF5	1.212 × 10^−12^	1.210 × 10^−12^	1.210 × 10^−12^	3.311 × 10^−20^	3.311 × 10^−20^	1.608 × 10^−2^	7.850 × 10^−3^	1.212 × 10^−12^
GF6	3.018 × 10^−11^	3.020 × 10^−11^	**2.060 × 10^−1^**	7.064 × 10^−18^	7.064 × 10^−18^	3.020 × 10^−11^	3.430 × 10^−6^	3.018 × 10^−11^
GF7	2.954 × 10^−11^	2.800 × 10^−11^	2.520 × 10^−5^	6.930 × 10^−18^	6.930 × 10^−18^	6.220 × 10^−11^	8.682 × 10^−3^	3.020 × 10^−11^
GF8	**1**	**3.337 × 10^−1^**	**1**	3.311 × 10^−20^	3.311 × 10^−20^	**1**	**1**	1
GF9	1.930 × 10^−11^	1.780 × 10^−11^	3.020 × 10^−11^	7.066 × 10^−18^	7.066 × 10^−18^	3.020 × 10^−11^	1.892 × 10^−4^	3.020 × 10^−11^
GF10	3.020 × 10^−11^	3.020 × 10^−11^	3.020 × 10^−11^	7.064 × 10^−18^	7.064 × 10^−18^	3.020 × 10^−11^	3.020 × 10^−11^	3.018 × 10^−11^
+/=/−	8/0/2	8/0/2	7/0/3	10/0/0	10/0/0	8/0/2	6/0/4	8/0/2

**Table 11:** Optimization results of robot gripper problem.

Algorithm	$x_{1}$	$x_{2}$	$x_{3}$	$x_{4}$	$x_{5}$	$x_{6}$	$x_{7}$	Result
WOA	100.64	31.39	100.00	0.00	10.00	100.00	1.00	3.43 × 10^−16^
HHO	149.93	94.19	106.04	39.01	55.00	183.57	2.77	9.52 × 10
SABO	146.50	115.87	171.86	19.45	143.92	181.63	2.95	5.52 × 10
OOA	130.03	78.89	146.72	48.38	102.96	150.36	3.03	1.16 × 10
RIME	146.95	146.62	191.93	0.13	145.98	106.44	2.39	2.82 × 10
RBMO	150.00	148.16	200.00	1.72	149.94	100.00	2.36	2.53 × 10
SWRBMO	100.00	38.19	200.00	0.00	10.00	100.00	1.44	**7.27 × 10^−17^**

**Table 12:** Optimization results of industrial refrigeration system design problem.

Algorithm	WOA	HHO	SABO	OOA	RIME	RBMO	SWRBMO
Result	25.88	16.61	9.10	7.80	7.91	7.73	**7.71**

**Table 13:** Optimization results of reinforced concrete beam design problem.

Algorithm	$x_{1}$	$x_{2}$	$x_{3}$	Result
WOA	8.000	29.689	7.867	1.65 × 10^2^
HHO	8.000	29.577	7.867	1.65 × 10^2^
SABO	8.000	30.967	8.006	1.72 × 10^2^
OOA	7.582	30.511	7.710	1.66 × 10^2^
RIME	7.999	29.623	7.869	1.65 × 10^2^
RBMO	8.000	29.954	7.867	1.65 × 10^2^
SWRBMO	8.000	29.390	7.867	**1.64 × 10^2^**

**Table 14:** Optimization results of step cone pulley problem.

Algorithm	$x_{1}$	$x_{2}$	$x_{3}$	$x_{4}$	$x_{5}$	Result
WOA	41.000	56.000	74.000	89.000	89.000	7.078 × 10^84^
HHO	41.000	56.000	75.000	90.000	90.000	6.806 × 10^80^
SABO	41.000	56.000	75.000	90.000	90.000	6.455 × 10^94^
OOA	43.000	56.000	85.000	87.000	87.000	6.265 × 10^97^
RIME	39.000	53.000	71.000	85.000	90.000	1.395 × 10^90^
RBMO	40.000	55.000	74.000	88.000	86.000	1.842 × 10^80^
SWRBMO	41.000	56.000	75.000	90.000	85.000	**1.712 × 10**

## Data Availability

Data are contained within the article.
